# Probiotic effects on immunity and microbiome in HIV-1 discordant patients

**DOI:** 10.3389/fimmu.2022.1066036

**Published:** 2022-12-08

**Authors:** Carlos Blázquez-Bondia, Mariona Parera, Francesc Català-Moll, Maria Casadellà, Aleix Elizalde-Torrent, Meritxell Aguiló, Jordi Espadaler-Mazo, José Ramon Santos, Roger Paredes, Marc Noguera-Julian

**Affiliations:** ^1^ IrsiCaixa AIDS Research Institute, Badalona, Spain; ^2^ AB-BIOTICS SA (Kaneka Group), Barcelona, Spain; ^3^ Infectious Diseases Department and Fundació Lluita contra les Infeccions, Hospital Universitari Germans Trias i Pujol, Badalona, Catalonia, Spain; ^4^ Centre for Health and Social Care Research (CESS), Faculty of Medicine, University of Vic – Central University of Catalonia (UVic – UCC), Vic, Barcelona, Spain; ^5^ Universitat Autònoma de Barcelona, Cerdanyola del Vallès, Spain; ^6^ Infectious Disease Networking Biomedical Research Center, Centro de Investigación Biomédica en Red de Enfermedades Infecciosas (CIBERINFEC), Carlos III Health Institute, Madrid, Spain

**Keywords:** probiotics, prebiotics, synbiotics, HIV, immune reconstitution

## Abstract

**Background:**

Some HIV-1 infected patients are unable to completely recover normal CD4+ T-cell (CD4+) counts after achieving HIV-1 suppression with combined Antiretroviral Therapy (cART), hence being classified as immuno-discordant. The human microbiome plays a crucial role in maintaining immune homeostasis and is a potential target towards immune reconstitution.

**Setting:**

RECOVER (NCT03542786) was a double-blind placebo-controlled clinical trial designed to evaluate if the novel probiotic i3.1 (AB-Biotics, Sant Cugat del Vallès, Spain) was able to improve immune reconstitution in HIV-1 infected immuno-discordant patients with stable cART and CD4+ counts <500 cells/mm3. The mixture consisted of two strains of *L. plantarum* and one of *P. acidilactici*, given with or without a fiber-based prebiotic.

**Methods:**

71 patients were randomized 1:2:2 to Placebo, Probiotic or probiotic + prebiotic (Synbiotic), and were followed over 6 months + 3-month washout period, in which changes on systemic immune status and gut microbiome were evaluated. Primary endpoints were safety and tolerability of the investigational product. Secondary endpoints were changes on CD4+ and CD8+ T-cell (CD8+) counts, inflammation markers and faecal microbiome structure, defined by alpha diversity (Gene Richness), beta diversity (Bray-Curtis) and functional profile. Comparisons across/within groups were performed using standard/paired Wilcoxon test, respectively.

**Results:**

Adverse event (AE) incidence was similar among groups (53%, 33%, and 55% in the Placebo, Probiotic and Synbiotic groups, respectively, the most common being grade 1 digestive AEs: flatulence, bloating and diarrhoea. Two grade 3 AEs were reported, all in the Synbiotic group: abdominal distension (possibly related) and malignant lung neoplasm (unrelated), and 1 grade 4 AE in the Placebo: hepatocarcinoma (unrelated). Synbiotic exposure was associated with a higher increase in CD4+/CD8+ T-cell (CD4/CD8) ratio at 6 months vs baseline (median=0.76(IQR=0.51) vs 0.72(0. 45), median change= 0.04(IQR=0.19), p = 0.03). At month 9, the Synbiotic group had a significant increase in CD4/CD8 ratio (0.827(0.55) vs 0.825(0.53), median change = 0.04(IQR=0.15), p= 0.02) relative to baseline, and higher CD4+ counts (447 (157) vs. 342(73) counts/ml, p = 0.03), and lower sCD14 values (2.16(0.67) vs 3.18(0.8), p = 0.008) than Placebo. No effect in immune parameters was observed in the Probiotic arm. None of the two interventions modified microbial gene richness (alpha diversity). However, intervention as categorical variable was associated with slight but significant effect on Bray-Curtis distance variance (Adonis R2 = 0.02, p = 0.005). Additionally, at month 6, Synbiotic intervention was associated with lower pathway abundances vs Placebo of Assimilatory Sulphate Reduction (8.79·10^-6^ (1.25·10^-5^) vs. 1.61·10^-5^ (2.77·10^-5^), p = 0.03) and biosynthesis of methionine (2.3·10^-5^ (3.17·10^-5^) vs. 4·10^-5^ (5.66·10^-5^), p = 0.03) and cysteine (1.83·10^-5^ (2.56·10^-5^) vs. 3.3·10^-5^ (4.62·10^-5)^, p = 0.03). At month 6, probiotic detection in faeces was associated with significant decreases in C Reactive Protein (CRP) vs baseline (11.1(22) vs. 19.2(66), median change= -2.7 (13.2) ug/ml, p = 0.04) and lower IL-6 values (0.58(1.13) vs. 1.17(1.59) ug/ml, p = 0.02) when compared with samples with no detectable probiotic. No detection of the probiotic was associated with higher CD4/CD8 ratio at month 6 vs baseline (0.718(0.57) vs. 0.58(0.4), median change = 0.4(0.2), p = 0.02). After washout, probiotic non-detection was also associated with a significant increase in CD4+ counts (457(153) vs. 416(142), median change = 45(75), counts/ml, p = 0.005) and CD4/CD8 ratio (0.67(0.5) vs 0.59(0.49), median change = 0.04 (0.18), p = 0.02).

**Conclusion:**

A synbiotic intervention with *L. plantarum* and *P. acidilactici* was safe and led to small increases in CD4/CD8 ratio and minor reductions in sCD14 of uncertain clinical significance. A probiotic with the same composition was also safe but did not achieve any impact on immune parameters or faecal microbiome composition.

## Introduction

One of the key aspects of HIV infection is a fast and widespread destruction of CD4+ T-lymphocytes (1), which becomes more exacerbated in the latest stages of infection (2, 3). Additionally, the virus presents the ability to establish reservoirs in which it can remain dormant mostly in high-CCR5 memory CD4+ T-cells (4, 5), where it remains integrated in the host genome (3–5). The ability to maintain a latency state has made it impossible to achieve a complete remission, although it can be life-long supressed in most patients with combined Antiretroviral Therapy (cART) (6). Nevertheless, even when the virus remains supressed, an important fraction of HIV-infected people will become immunodiscordant, as they fail to fully recover CD4+ counts and immune function (7–9) especially those who failed to receive cART on the early stages of infection.

This lack of recovery stems from the virus early replication site and its reservoir sanctuary: the Gut-Associated Lymphoid Tissue (GALT) (10), where the biggest population of high CCR5+ CD4+ T-cells resides. Depletion of such cells in the gut is coupled with a decrease in Treg numbers (11), but more slowly than its CD4+ counterpart, lowering the ratio between Treg and effector CD4+ cells, especially the Th17 subtypes (11, 12). A relative decrease of the Th17/Treg ratio has been correlated to a higher chance of disease progression (13). Th17 have also been shown to maintain gut barrier integrity, stimulating tight junction expression in epithelial cells (14), as well as modulating bacterial populations in the mucosa by secreting antimicrobial peptides (15). Hence, the skew in the Treg/Th17 ratio compromises the gut barrier integrity, and creates a feedback loop where dysbiosis and gut inflammation cause a leakage of bacterial compounds into the bloodstream (16), which increases residual systemic inflammation (17) and leads to further T-cell exhaustion and senescence (18), which further promotes dysbiosis and gut barrier. This vicious circle ends up causing immune exhaustion and may hinder any attempt of immune reconstitution.

While many approaches have been considered to recover immunity and gut integrity, modulation of the gut microbiome awakened great interest recently. It is now known that some bacterial species directly affect the immunologic makeup of the gut barrier (19) by modulating tryptophan to kynurenine catabolism (17). This pathway is thought to promote Treg differentiation (17) and has been shown to increase with presence of some *Proteobacteria* (20) and decrease with *Lactobacillus species (* 20, 21). For this, probiotics have been widely tested with promising results for wide variety of ailments, both from the gut itself such as Inflammatory Bowel Disease (IBD) (22), diarrhoea, both HIV-induced (23) and by other pathogens (24) and even outside of the gut, such as allergies and upper respiratory infections (25).

Recently, the gut microbiome has awakened a great interest in the scope of HIV clinical management, as immune recovery has been shown to be affected by the state of the gut mucosa (26). Cross-sectional studies have found microbiome signatures correlated to immune reconstitution such as higher Prevotella/Bacteroides ratio and enrichment of *Faecalibacterium prausnitzii and Coprococcus comes* (27) or increased abundance of *Fusobacterium* negatively linked to immune recovery (28). Consequently, probiotic interventions have have become an interesting therapeutic target, as they promote tolerogenicity (29), displace pathogenic strains, and reduce inflammation (30), which could reduce T cell depletion and senescence, opening a way to improve immune reconstitution after viral suppression. However, many different combinations of probiotic strains and prebiotic substrates have been tested with mixed results (30, 31).

In this study, we performed a randomized double-blinded trial to test the safety, tolerability and effectiveness of a probiotic consisting of two strains of *Lactobacillus plantarum* and one of *Pediococcus acidilactici*, combined with prebiotic fibers over the course of 6 months, followed by a 3-month long washout period in immunodiscordant (<500 counts/ml) HIV patients with stable cART. The primary endpoints consisted of safety and tolerability. The secondary endpoints were changes in CD4+, CD8+ counts, CD4/CD8 ratio, inflammation, and gut permeability markers, as well as changes in the gut microbiome taxonomical and functional composition after 6 months of intervention + 3 of washout.

## Materials and methods

### Ethics statement

The study was reviewed and approved by the Institutional Review Board of the Hospital Universitari Germans Trias i Pujol (reference PI-13-046). All participants provided written informed consent in accordance with the World Medical Association Declaration of Helsinki, Fortaleza and Brazil, October 2013 and personal data was managed according to Spanish data protection law (LOPD 15/1999). The study concept, design, patient information and results were discussed with the FLSida Community Advisory Committee, in accordance with AB-Biotics internal QC auditing. All available information can be found in the protocol (**Supplementary Methods**), and the study is registered in clinicaltrials.gov, accession: NCT03542786.

### Cohort description

This study took place in a two-year span between 2017 and 2019 and was designed as a masked randomized, placebo-controlled, double-blinded three-arm study in a cohort of 100 HIV+ patients with the following inclusion criteria: 18 years of age or older, chronic HIV infection with stable Anti-Retroviral Treatment (cART) ongoing for longer than a year prior to the start, peripheral CD4+ counts lower than 500 cells/ml in plasma, <50 HIV copies/ml in plasma for at least 6 months before the start, no antibiotic treatments at least 1 month before start, lack of severe AIDS-defining diseases and no pregnancy. An additional filter was later implemented, in which only those patients with at least 2 samples along the trial would be selected for further analysis, to preserve the longitudinal approach of the study.

After inclusion, patients were randomly assigned to one of the three following groups: Placebo, Probiotic or Synbiotic in a 1:2:2 ratio and matched by 3^rd^ cART drug class: Integrase Strand Transfer Inhibitors (INSTI), Non-nucleoside reverse transcriptase inhibitors (NNRTI) or Protease Inhibitors (PI), and CD4+ nadir higher or lower than 200 cells/ml at the time of screening.

All recruited patients followed a 6-month treatment followed by a 3-month washout periods, with check-ups at months 0,1,3,6 and 9. Every follow-up visit consisted of a sample collection of both blood and faeces (except at the 1^st^ month checkout, where only stool was collected), a physical examination and a questionnaire about quality of life, and self-reported treatment adherence since last check-up. During the treatment period, all participants received different formulation depending on whether they received a prebiotic + probiotic (Synbiotic), probiotic alone (Probiotic) or a placebo (Placebo), which was administered orally as dissolved powder sachets, daily.

### Treatment formulations

The probiotic used in this study consisted of a mix of 3 *Lactobacillales* strains: *L. plantarum* (strains CECT7484 and CECT7485) and *P. acidilactici* (strain CECT7483). In the Synbiotic group, the probiotic was co-administered with two different mixes of vegetal fibers consisting of pectin, inulin, oat, acacia, maltodextrin polydextrose and Partially Hidrolyzed Guar Gum (PHGG) that were alternatively combined with the probiotic every other week (the exact formulation can be found in the protocol). The Probiotic and Placebo groups received excipient-containing envelopes that were identical to those of the Synbiotic group to preserve the double-blind. The exact composition and manufacturing process can be found in **Supplementary Methods**.

### T-cell and inflammation marker quantification

Blood samples were collected in fasting conditions at the same time as stool samples, when possible. A fraction of these samples was used as whole blood to perform peripheral CD4+ and CD8+ counts by flow cytometry at the Germans Trias i Pujol Hospital. Soluble markers of microbial translocation in plasma (sCD14 and LBP) and inflammation markers (IL-6, D-Dimer and CRP) were quantified using sCD14 and LBP DuoSet ELISA development system (pg/mL), R&D systems (Minneapolis, MN), Human IL6- High Sensitivity ELISA, Invitrogen (Waltham, Massachusetts, USA) and RayBio Human D-Dimer or CRP Elisa Kit, RayBiotech (Peachtree Corners, GA, USA), respectively.

### Faecal DNA extraction, library preparation and sequencing

Stool samples were collected by the patient or nursing staff according to the GUT (DNA Stabilized-frozen Inc., Ottawa, Ontario, Canada) extraction kit. Samples were stored at -80°C till processing. Faecal DNA was extracted using the PowerSoil DNA Extraction Kit (MO BIO Laboratories, Carlsbad, CA, USA), which was then fragmented into 300 bp clone-sized libraries using Nextera-XT Illumina kit (Illumina, Inc. San Diego, CA, USA) and sequenced in an Illumina HiSeq sequencer (Illumina, Inc. San Diego, CA, USA) with a sequencing depth target of 20 million reads.

### Sequence filtering and quality control

Raw.*fastq* files were first processed for quality control. Read Quality filtering and trimming was performed with trimmomatic (32), with a 30-nt sliding window approach, trimming when the average phred score dropped below 20. Trimmed reads were then aligned against the human Hg19 genomic database using bowtie2 to remove any human DNA contamination.

### Taxonomy annotation

Taxonomy assessment was performed with Metaphlan3 (33), performing a marker gene-based quantification, using the CHOCOPhlan 201901.1 database. The obtained data was packaged into an R phyloseq structure.

### Gene function and pathway diversity analysis

Parallel to taxonomic analysis, gene function and metabolic pathway quantification was performed from the raw, quality-filtered sequencing data using HUMAnN3.0 (33). The software was run with its standard configuration and built-in databases. Results from all samples were combined into a unique table using HUMAnN3 inner script *merge_tables.sh* and clustered into Metacyc pathway abundances.

### Alpha and beta diversity metrics

In this study, ecological alpha diversity was studies as gene richness. To obtain it, the post-QC sequencing data was aligned against the Integrated Gene Catalog (IGC) database (34) using Bowtie2 (35). The output was sampled at different numbers of reads to obtain rarefaction curves, from which the minimum sampling threshold was defined as the 95^th^ percentile of maximum per-sample coverages, which equalled 2·10^7^ reads. Samples with max coverages below this threshold were discarded, and those above were subsampled to said value to remove coverage biases.

For beta diversity, the taxonomy tables were used to construct pairwise Bray-Curtis distance matrixes between samples. The obtain matrices were then projected into NMDS coordinates using the function MetaMDS from the R vegan (36) package using their default configuration.

### Statistical testing

All statistical testing was performed using R 4.0.2. Since most of the quantitative variables tested departed from normality, assessed by Shapiro-wilk test, comparisons across group were performed using Wilcoxon Rank-Sum test, while within-group, longitudinal comparisons were performed using Wilcoxon rank-sum matched-pairs test, with Benjamini-Hochberg correction. In the case of gene pathways, Kruskal-Wallis tests between groups were performed to filter out those pathways which changed in any timepoint other than basal, before proceeding to the pairwise group/timepoint comparisons using a p < 0.05 cut-off. Statistical results are reported as median (IQR), and longitudinal tests included median change (IQR). The number of patients per group for each statistical comparison performed in this study can be found in **Supplementary Tables 3**, **4**, for categorical and longitudinal comparisons, respectively.

In addition, we studied the changes in time for numerical variables (diversity, inflammation, T-cells, and pathways) with Linear Mixed Models (LMM) using the Lme4 package. Models were built in two different approaches: one where the dataset was split by group, in which the LMM would be built for each using time as the only fixed effect, in order to study the magnitude of the change for each group and its significance, and another where an LMM was used with the entire dataset with both group and time as fixed effects, to assess the difference in change in slope between groups and its significance *via* two-way ANOVA. In all cases, the patients individual IDs were inputted as the random variable.

Bray Curtis distances were compared between groups using PERMANOVA. T-cell counts, inflammation and gut permeability markers were correlated with the NMDS coordinates using Spearman correlation.

## Results

### Cohort description and follow-up

**Figure 1:**
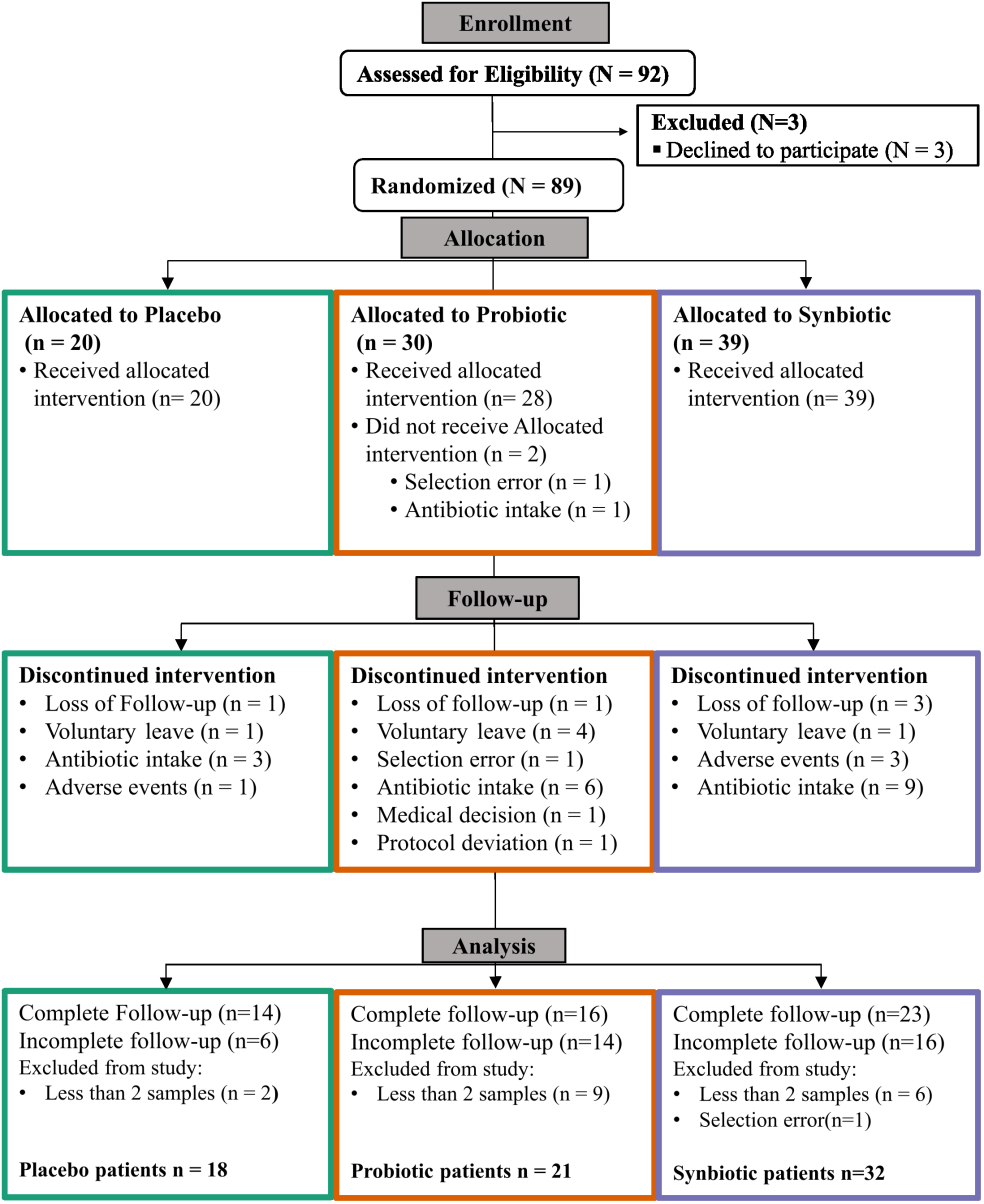
CONSORT (Consolidated Standards of Reporting Trials) Flow diagram.

From the original goal of 100 patients recruited of which 92 passed all the inclusion criteria, 89 patients were successfully randomised for the study. At study ending, 53 patients had complete follow-up and 36 had incomplete follow-up, of which 18 provided samples for at least two timepoints. Thus, 71 patients were finally selected for analysis, resulting in a proportion of 18 patients in the Placebo, 21 in the Probiotic and 32 in the Synbiotic groups (**Figure 1**). The self-reported mean adherences to the treatment showed no significant differences between groups (96% for the Placebo, 89.7% for Probiotic and 96.6% for the Synbiotic group). Demographic and clinical variables (**Table 1**, **Supplementary Table 1**) were tested with the corresponding statistical method depending on their normality according to a previous Shapiro test.

**Table 1:** Study population description of demographic variables and clinical markers at baseline: Age, height, LBP, and weight represented as mean-(sd), as they followed a continuous normal distribution (Shapiro test pval < 0.05).

		[ALL]	Placebo	Probiotic	Synbiotic	p.overall	N
		*N = 71*	*N = 18*	*N = 21*	*N = 32*		
Age		49.9 (9.36)	52.8 (10.4)	49.1 (10.4)	48.8 (7.88)	0.316	71
CD4+ nadir		124 [74.5;236]	140 [81.8;212]	109 [74.0;186]	157 [70.0;261]	0.395	71
Gender:	F	10 (14.1%)	1 (5.56%)	5 (23.8%)	4 (12.5%)	0.310	71
	M	61 (85.9%)	17 (94.4%)	16 (76.2%)	28 (87.5%)		
BMI		24.4 [22.1;25.6]	24.8 [22.6;26.3]	24.4 [22.7;25.3]	24.1 [22.2;25.4]	0.778	69
Weight (Kg)		72.8 (11.0)	75.2 (12.6)	72.8 (12.3)	71.3 (9.08)	0.491	69
Height (cm)		172 (7.13)	174 (9.26)	171 (6.98)	171 (5.71)	0.336	71
Third drug class	INSTI	47 (66.2%)	9 (50.0%)	16 (76.2%)	22 (68.8%)	0.282	71
	NNRTI	18 (25.4%)	6 (33.3%)	5 (23.8%)	7 (21.9%)		
	PI	6 (8.45%)	3 (16.7%)	0 (0.00%)	3 (9.38%)		
LBP (μg/mL)		5.99 (2.17)	5.71 (2.31)	6.12 (2.54)	6.06 (1.86)	0.815	70
sCD14 (μg/mL)		2.48 [2.01;2.92]	2.62 [2.03;2.95]	2.62 [2.03;3.25]	2.36 [1.90;2.61]	0.087	70
IL6 (pg/ml)		0.95 [0.65;1.72]	0.87 [0.59;2.41]	0.74 [0.64;1.67]	1.02 [0.68;1.42]	0.934	70
CRP (μg/mL)		18.1 [10.0;64.5]	17.5 [9.14;48.0]	15.3 [13.4;56.9]	20.3 [10.4;65.6]	0.901	70
D-Dimer (μg/mL)		2.76 [2.14;3.69]	2.67 [2.02;3.96]	2.56 [1.97;3.48]	2.76 [2.39;4.02]	0.290	70
CD4+ (counts/mL)		397 [337;466]	358 [344;427]	403 [318;466]	422 [338;476]	0.259	68
CD8+ (counts/mL)		696 [507;870]	734 [603;819]	690 [507;877]	657 [500;865]	0.583	68
CD4/CD8		0.55 [0.42;0.83]	0.49 [0.42;0.61]	0.56 [0.40;0.67]	0.57 [0.45;0.88]	0.333	68

Age, weight, height, and LBP and were tested by ANOVA, categorical variables were compared using Fisher’s exact test, while non-normal continuous variables (BMI, CD4 + nadir, years with cART, diagnosis, and other clinical variables) were tested by Kruskal-Wallis. Third drug row refers to the class of the 3^rd^ ARV drug they received INSTI, Integrase Strand Transfer Inhibitor; NNRTI, Non-nucleoside reverse transcriptase inhibitors. All other variables represented as either count (percentage), or in the case of non-normally distributed as median-[Q1;Q3].

### Safety and adverse events

The proportion of patients who suffered at least one Adverse Event (AE) during the study remained comparable between all arms, with no significant differences found: 9 patients with AE (50%) in the Placebo, 7 (33%) in the Probiotic and 17 (53%) in the Synbiotic groups (**Supplementary Table 2**). Most AEs consisted of severity grade 1 and 2, being the gastrointestinal conditions the most frequent, especially flatulence, dyspepsia, and diarrhoea, although other conditions were reported, but could not be associated with treatment group assignation. Two instances of severe grade 3 AEs were reported: abdominal distension (possibly related) and malignant lung neoplasm (unrelated), cancer (unrelated), while one grade 4 event (hepatocarcinoma, unrelated) was reported in the Placebo group.

### Synbiotic formulation does not affect CD4+ and CD8+ counts but correlates with increased CD4/CD8 ratio and reduced inflammation

**Figure 2:**
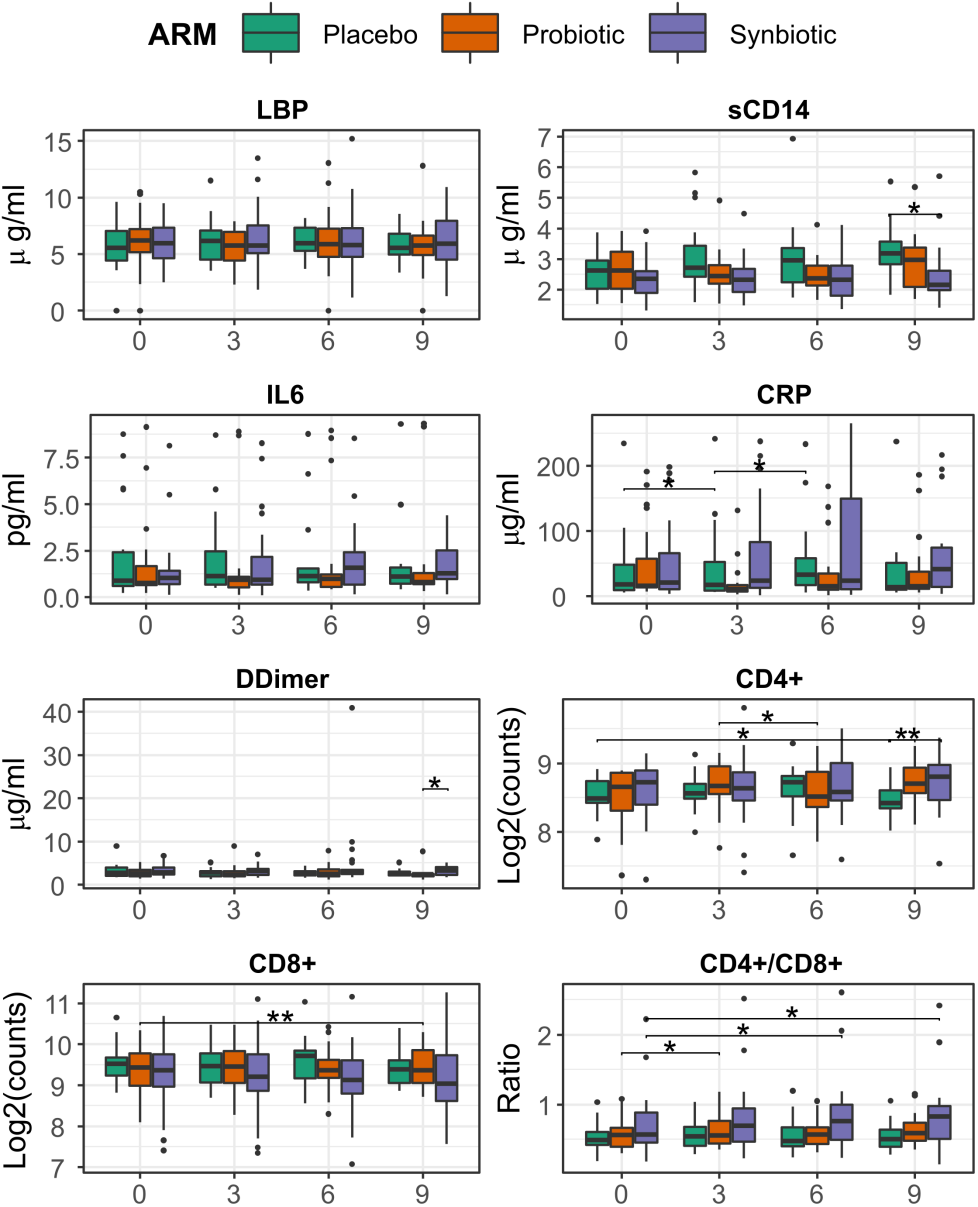
Evolution of T-cell counts, ratio, inflammation, and bacterial translocation markers between months 0 and 6, among treatment group. Comparisons within group and between time points were performed by Wilcoxon test, in its paired form for longitudinal differences and unpaired for cross-group comparisons. Significance was coded as follows: *(p < 0.05), **(p < 0.01).

CD4/CD8 ratio showed a slight albeit significant increase in the Synbiotic group at month 6 respect to baseline (median=0.76 (IQR=0.51) vs 0.72 (0.45), median change= 0.04 (0.19), p = 0.03). At month 9, the Synbiotic group still had increased CD4/CD8 vs baseline (0.827 (0.55) vs 0.825 (0.53), median change = 0.04 (0.15), p= 0.02), CD4+ was higher (447 (157) vs 342 (73) counts/ml, p = 0.03), and sCD14 was lower (2.16 (0.67) vs 3.18 (0.8) p = 0.008) than Placebo (**Figure 2**).

Analysis with LMMs mirrored most of these previous findings, a significant increasing trend was found for both CRP (ANOVA p = 0.049, slope = 4.5) and CD4/CD8 ratio (p = 0.002, slope = 0.012) in the Synbiotic group (**Supplementary Figure 2**).

Finally, CD8+ counts appeared to be impacted by overall microbiome structure, as it was positively correlated with the 2^nd^ coordinate of the NMDS (**Supplementary Figure 1**) and, as a result, CD4/CD8 ratio was negatively correlated (**Supplementary Table 5**)

### Intervention doesn’t correlate with faecal microbiome changes

**Figure 3:**
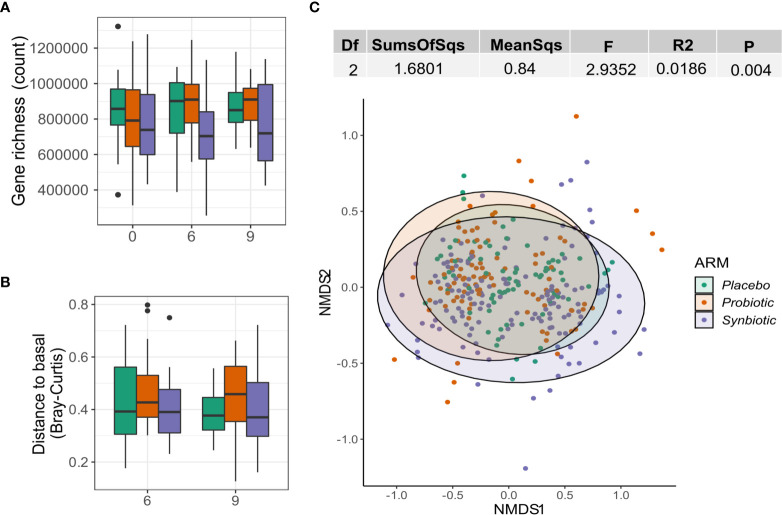
Effect of probiotics on the gut microbiome. **(A)** Gene richness counts for each intervention arm and month. Comparisons between group at each timepoint were performed using Wilcoxon test, while the paired version was used for within-group comparisons between months. **(B)** Distribution of Bray Curtis-Distances from baseline for each month and intervention arm. **(C)** NMDS showing sample ordination according to Bray-Curtis distance. Group distance dissimilarity was tested using ADONIS.

No significant differences in gene richness were detected either between groups or longitudinally within groups (**Figure 3A**). Beta diversity did not increase either, as no changes in Bray Curtis distance vs each patient’s respective baseline could be observed (**Figure 3B**). NMDS showed no significant clustering based on Bray-Curtis distance and the intervention variable only explained 1.8% of the variance (Adonis R2 = 0.02, P=0.0051; **Figure 3C**).

### Synbiotic supplementation affects sulphate assimilation on the gut

**Figure 4:**
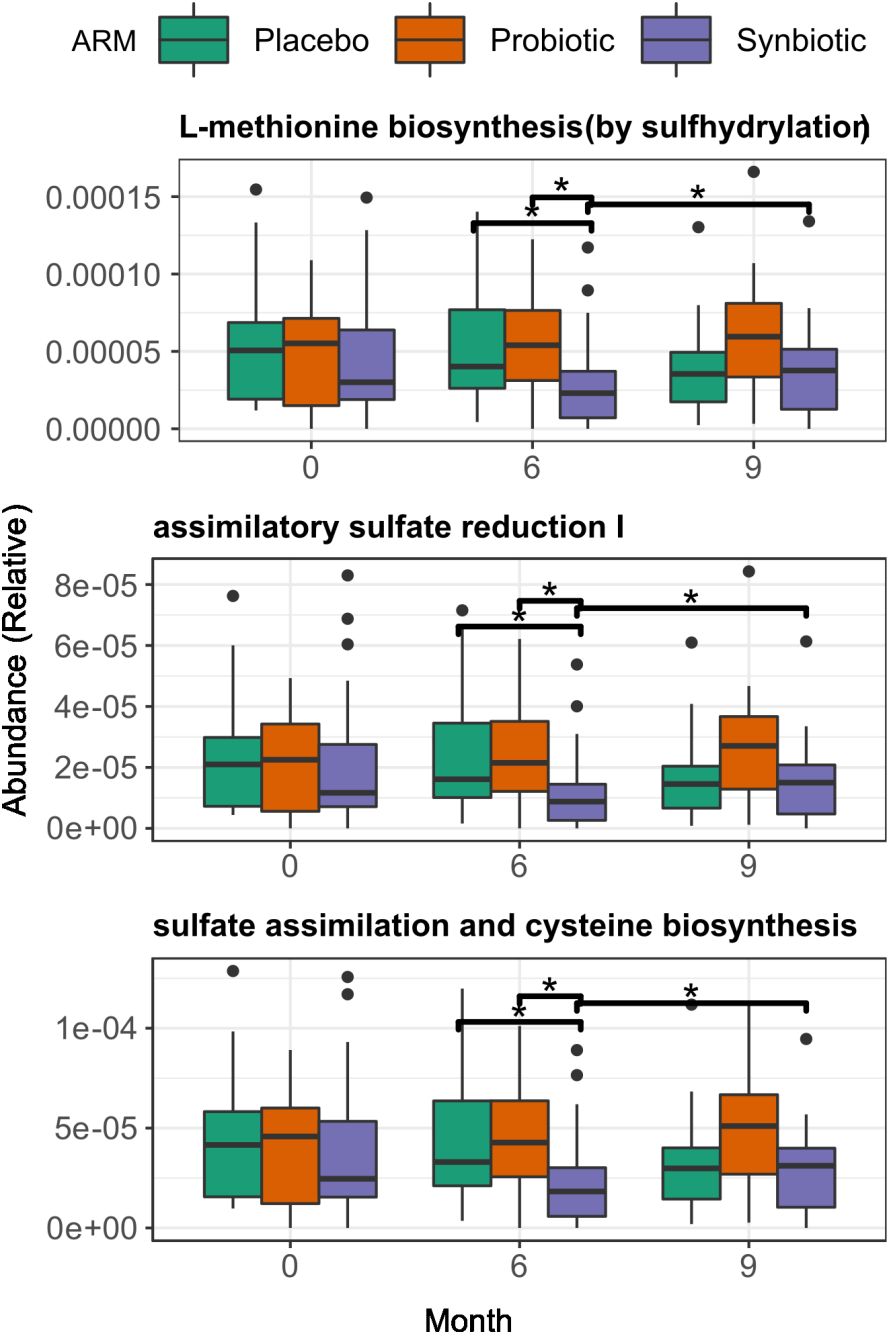
Changes in gene function at both start and end of the treatment period, among treatment group. Across group comparisons were performed with regular Wilcoxon test and longitudinal comparisons were performed using matched-samples Wilcoxon test. Significance was coded as follows: *(p < 0.05).

An exploratory analysis between groups identified 3 biochemical pathways with significant changes in relative abundance at month 6 (Kruskal-Wallis p < 0.01). Those pathways were consistently lower in the Synbiotic group respective to the Placebo: L-methionine biosynthesis (Met) (2.3·10^-5^ (3.17·10^-5^) vs 4·10^-5^ (5.66·10^-5^), p = 0.03), Assimilatory Sulphate Reduction (ASR) (median = 8.79·10^-6^ (IQR=1.25·10^-5^) vs 1.61·10^-5^ (2.77·10^-5^), p = 0.03), and cysteine biosynthesis (Cys) (1.83·10^-5^ (2.56·10^-5)^ vs 3.3·10^-5^ (4.62·10^-5^), p = 0.03). At month 9, all three pathways increased in the Synbiotic group vs month 6 (Met: 3.8·10^-5^ (4.2·10^-5^) vs 2.3·10^-5^ (3.17·10^-5^), median change = 1.4·10^-5^ (3.12·10^-5^), p = 0.03; ASR: 1.5·10^-5^ (1.7·10^-5^) vs 8.79·10^-6^ (1.3·10^-5^) median change = 5.6·10^-6^ (1.3·10^-5^); p = 0.03; Cys: 3.1·10^-5^ (3.2·10^-5^) vs 1.8·10^-5^ (2.6·10^-5^), median change= 1.13·10^-5^ (2.42·10^-5^), p = 0.03), but no significant changes were found in the other groups (**Figure 4**).

A more in-depth approach with LMMs suggested a significant decreasing trend in the relative abundances of all three pathways over time in the Synbiotic group (Met: p= 0.003, slope = -3.12·10^-6^; ASR: p = 0.003, slope = -1.59·10^-6^; Cys: p = 0.003, slope = -2.53·10^-6^) (**Supplementary Figure 4**). Additionally, a test of fixed effect interaction found a significant effect of intervention over the magnitude of change over time of pathway relative abundance (ANOVA = 0.008, **Supplementary Figure 6A**).

### Presence of probiotic strains in faeces does not relate to changes in faecal microbiome

**Figure 5:**
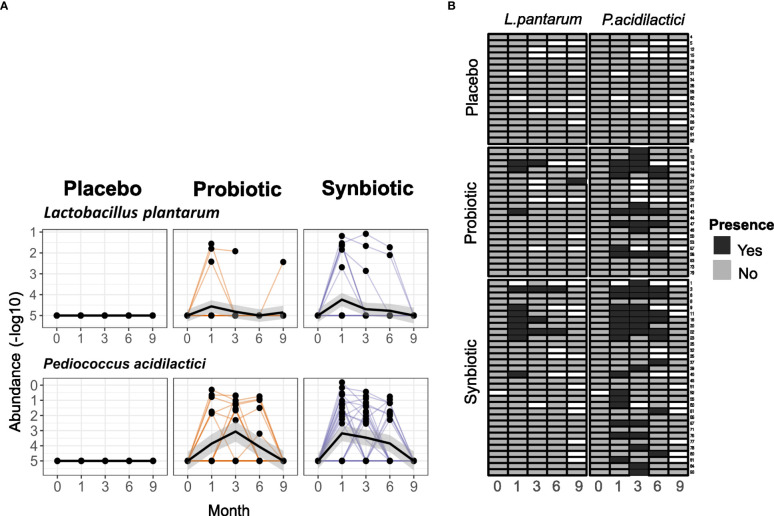
Detection of the probiotic strains in stool samples. **(A)** Relative abundances (in -log10) of *P. acidilactici* and *L. plantarum* in faeces for each of the intervention arms. **(B)** Heatmap representing presence/absence of each arm in each sample. Rows represent single patients and columns represent the month each sample belongs to. White cells represent lack of sample.

To better understand the lack of microbiome changes and the uncertain clinical changes, we tested how well the probiotic strains could maintain their presence in the gut. Only 6 (28.6%) patients in the Probiotic and 10 (31.3%) in the Synbiotic groups had detectable levels of either *L. plantarum* or *P. acidilactici* in at least two stool samples at months 1, 3 or 6, although in these cases, probiotic species median relative abundance started declining after the 3^rd^ month of treatment, long before the wash-out phase. In the remaining patients, probiotic species were detected in only one of the timepoints in 5(23.8%) and 13(40%) of the Probiotic and Synbiotic, respectively. None of the probiotic strains was detected in any sample obtained at baseline, month 9 or in the Placebo group at any time point (**Figure 5A**).

To better filter any potential probiotic strain-specific effect, as well as uncover potential factors that may affect probiotic engraftment, a new variable was defined. Two groups were created that separated patients with or without detectable levels of *P. acidilactici* in at least 2 stool samples belonging to different time points, excluding those patients in the Placebo group. *P. acidilactici* was chosen because it had presence in all samples where *L. plantarum* was detectable, but not the other way around (**Figure 5B**).

**Figure 6:**
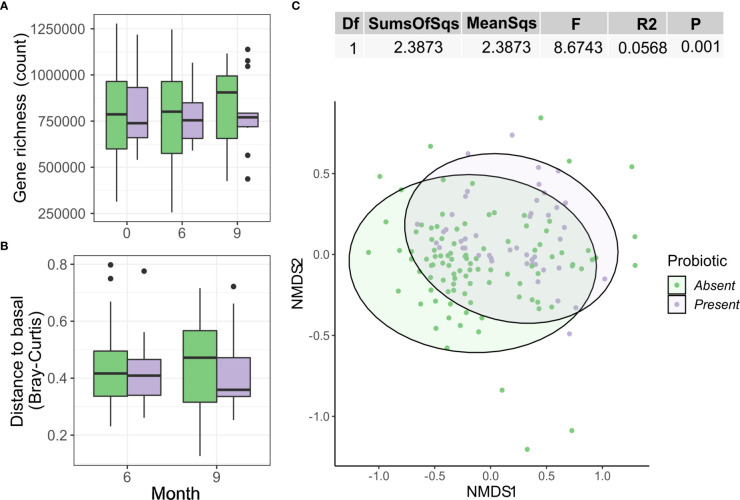
Differences by presence or absence of the probiotic strains on the gut microbiome. **(A)** Gene richness counts for each group arm and month. Comparisons between group at each timepoint were used simple Wilcoxon test, while the paired version was used for within-group comparisons between months. **(B)** Distribution of Bray Curtis-Distances from baseline for each month and group. **(C)** NMDS showing sample ordination according to Bray-Curtis distance. Group distance dissimilarity was tested using ADONIS.

Microbiome analysis under this new stratification found no significant changes in alpha diversity as gene richness (**Figure 6A**) or beta diversity as Bray-Curtis distance from baseline (**Figure 6B**) over time between groups with (Present) and without (Absent) probiotic detection. This new variable only explained a 3.6% of the variance of Bray-Curtis distances between samples (Adonis R2 = 0.0362, p = 0.001). No clear group clustering could be observed under this new definition (**Figure 6C**)

### Detection of probiotic in faeces associates with decreased inflammation (CRP, IL-6) while non-detection associates with increased CD4/CD8 ratio

**Figure 7:**
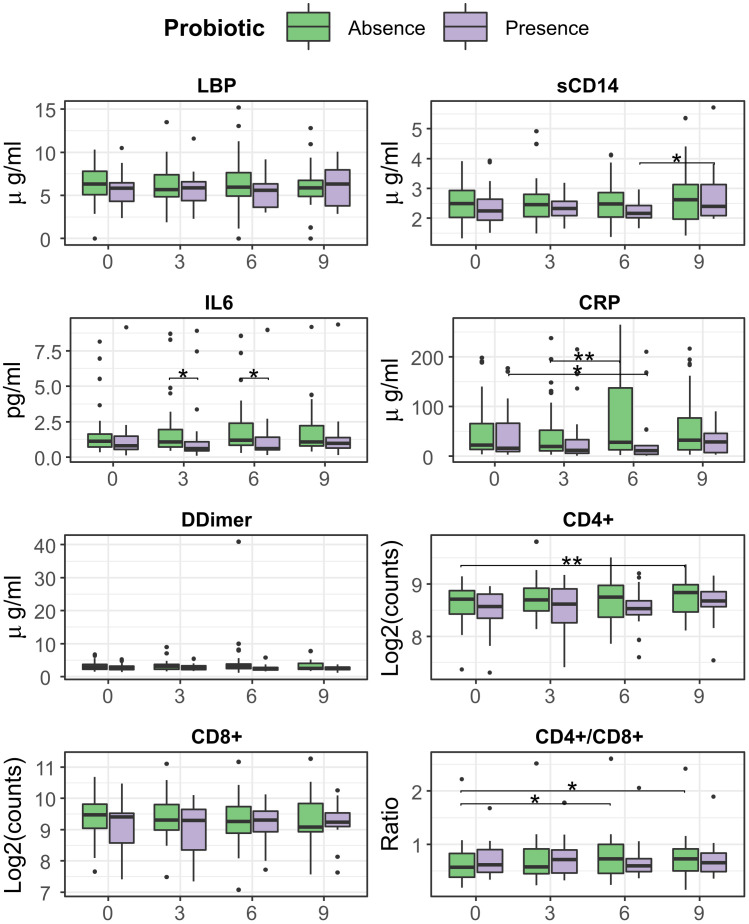
Evolution of T-cell counts, ratio, inflammation, and bacterial translocation markers between months 0 and 6, separated presence/absence of probiotic strains. Comparisons within group and between time points were performed by Wilcoxon test, in its paired form for longitudinal differences and unpaired for cross-group comparisons. Significance was coded as follows: *(p < 0.05), **(p < 0.01).

At month 6, the Present group was associated with a significant decrease of CRP (median=11.1 (IQR=22) vs 19.2 (66), median change= -2.7 (13.2) ug/ml, p = 0.04), while the Absent group related to an increase in CD4/CD8 ratio (0.72 (0.57) vs 0.56 (0.4), median change = 0.4 (0.2) p=0.015) vs baseline. IL-6 differed between both groups at month 6, being lower in Present than Absent group (0.58 (1.13) vs 1.17 (1.59) ug/ml, p = 0.02). At month 9, an increase vs baseline was observed for CD4/CD8 ratio (0.67 (0.5) vs 0.59 ± (0.49), median change = 0.04 (0.18), p = 0.02), and CD4+ counts (457 (153) vs 416 (142), median change = 45 (75), counts/ml, p = 0.005) in Absent but not in Present group (**Figure 7**).

Analysis with LMM found little overall effect on immune status and inflammation, with a slight increase of CD4/CD8 ratio (p = 0.008, slope = 0.012) and D-Dimer (p = 0.047, slope = 0.236) in the Absent group (**Supplementary Figure 3A**). However, these trends changed when stratifying by treatment group, especially for CRP, which increased in the Synbiotic arm (p = 0.013, slope = 7.4) (**Supplementary Figure 3C**). Also, CD4/CD8 ratio increase was specific to the subjects in Absent group who were in the Synbiotic arm (p = 0.005, slope = 0.015).

### Probiotic presence in faeces is not coupled with lower abundances of ASR pathways

**Figure 8:**
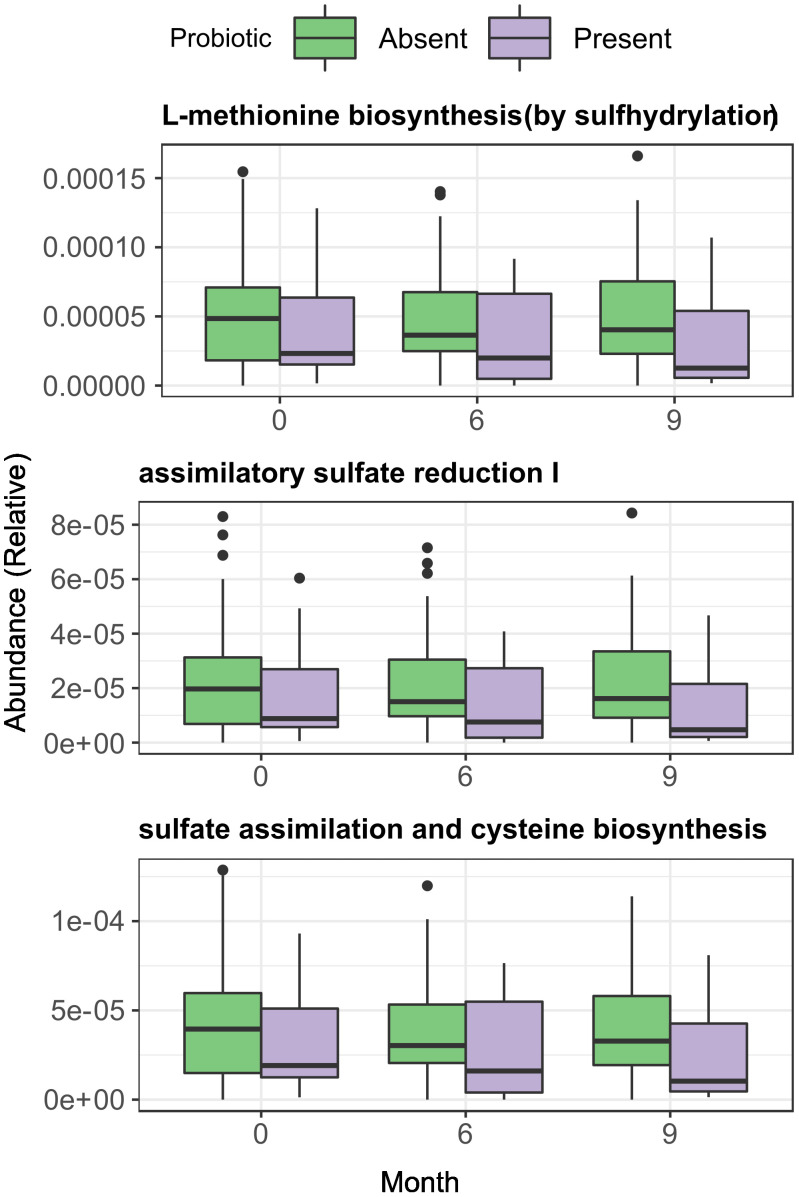
Longitudinal and across-group comparisons of Assimilatory Sulphate Reduction and sulphur amino acid pathways across intervention groups. Comparisons were performed across between groups by Wilcoxon test and longitudinally within-group with paired Wilcoxon test.

Sulphate assimilation pathways seemed slightly reduced in the probiotic Present vs Absent group, but the differences were statistically non-significant (**Figure 8**). Analysis with LMMs found no significant trends for any of the three previously studied pathways neither in the Present nor in the Absent group. Interestingly, after stratifying by intervention, pathway relative abundances did not change over time in the Present patients within the Probiotic group but increased in the Absent group (Met: p = 0.026, slope = 2.13·10^-6^; ASR: p=0.024, slope = 1.11·10^-6^; Cys: p = 0.021, slope = 1.256·10^-6^), while the Synbiotic group showed a relative abundance decline in both Present and Absent groups (**Supplementary Figure 5**). Fixed effect interaction tests on unified models found no significant effect of presence/absence in any of the pathways, with and without stratification (**Supplementary Figure 6**).

## Discussion

We assessed the efficacy and safety of long-term probiotic supplementation on immunodiscordant (CD4+ counts < 500) patients with supressed HIV after long, stable cART in a double-blind, randomized trial. We found that Synbiotic intervention with strains of *L. plantarum* and *P. acidilactici* along with prebiotic fibers is safe and associated with slight increases of CD4+ counts, CD4/CD8 ratio, and a decrease of the gut leakiness, as measured with sCD14 which is a proxy for bacterial endotoxin entering the bloodstream. However, the clinical significance of such improvements is uncertain, especially as they manifested 3 months after the intervention stopped. A more in-depth analysis suggests that the presence of the probiotic strains in stool was associated with lower levels of proinflammatory cytokines (IL-6 and CRP), while improvements in CD4/CD8 ratio appeared to be linked to the prebiotic fibers. The lowering in proinflammatory cytokines seems in agreement with previous reports using the same probiotic composition in animal models of IBD (37). This could point to a trade-off between immune activation and modulation by the prebiotic and probiotic, respectively. Of note, the reduction in inflammation markers happened during the intervention period, when no changes in gut permeability markers could be observed, suggesting that the immunomodulatory activity of the probiotics may be independent of the gut barrier status. Some gut barrier-unrelated anti-inflammatory mechanisms have been described, as Kawashima, Tadaomi et al. (38) found, lactic acid bacteria can induce IgA secretion to the gut lumen, which is a known immunomodulatory agent (39, 40).

The health-promoting properties of lactic acid bacteria have been widely reported *in vivo* and *in vitro* (41, 42). Nevertheless, the actual implantation and ecological viability of such strains is a multidimensional problem where host related factors and inherent ecological features of the host microbiome (such as normal ecological succession across the intestine) (43). Within the gastrointestinal (GI) tract, some lactobacilli show a nomadic behaviour (44) and tend to stay most commonly on the upper GI hence they tend to be underrepresented and transient in stool samples (45). In turn, implantation itself is not a strict requirement to generate change in the gut ecosystem or the host’s physiology (46). In this study, *P. acidilactici* was more consistently found than *Lactobacillus plantarum* in faeces but, unlike the later, the former has been reported as having good adhesion to the lower GI and higher presence in stool (47). Noteworthy, such differences may be strain-specific and not generalizable to other formulations (43). Hence, assessing the effectiveness of the probiotic strains in this study has been problematic, due to the low proportion of patients having a detectable presence of those, but also to the spatial and temporal complexity that characterizes the gut microbiome.

Additionally, we found that synbiotic intake was linked to decreases in the Assimilatory Sulphate Reduction (ASR) and sulphur-containing amino acids biosynthesis pathways. ASR, unlike the Dissimilatory Sulphate Reduction (DSR), takes sulphate without producing hydrogen sulphide as a final product, which has been shown to impair butyrate oxidation, the primary source of energy of enterocytes, and has been linked to gut inflammation and Ulcerative Colitis (48, 49). DSR is exclusive of anaerobic bacteria that undergo sulphate respiration, while ASR is more ubiquitous (50). Intake of Fructose and Glucose Oligosaccharides (FOS and GOS respectively) has been extensively used to modulate the microbiome, and has been linked to metabolic changes, especially of short chain fatty acids (51), but little is known about their relationship with sulphate metabolism. The fact that the Synbiotic group showed a consistent decline in such pathways could point to metabolic modulation and a population shift by the prebiotic fibers in the small intestine, although such changes may have been partially represented in the faecal samples.

Previous studies also found a lack of change in neither peripheral blood T-cells and/or gut permeability markers with probiotics only. Serrano-Villar et al. (52) found a significant decrease in inflammation markers (IL-6 and CRP) but no differential improvement in either circulatory T-cells nor sCD14 after 48 weeks, using a synbiotic formulation of *Saccharomyces boulardii* with various additives. However, this study was performed on late presenter, cART-naïve individuals, while our study was conducted on immunodiscordant patients with stable cART.

Previous studies using HIV infected immune discordant cohort reported diverse results. Presti et al. (53) tested a probiotic treatment consisting of different strains of streptococcus, bifidobacteria, and lactobacilli, without prebiotics, over 12 weeks, finding no differences in gut integrity (sCD14) and inflammation markers (D-Dimer, IP-10) but a significant decrease in *Proteobacteria*. Stiksrud et al. (54) found a decrease in inflammation (IL-6 and D-Dimer) but no differences in bacterial translocation markers and CD4+ counts after 8 weeks of intervention with formulation of skimmed milk, enriched with various species of *Lactobacillus* and Bifidobacterium vs a placebo of skimmed-milk only and a control groups. Geng et al. (55) reported an improvement in gut integrity (D-Dimer, DAO) and an enhanced CD4+ recovery in immune discordant patients using pre-digested protein supplementation. Despite the differences from the previously described studies (whether from study design or probiotic formulation), our study found improvement in inflammation and translocation markers but not an overall improvement in immune reconstitution. In addition, we could assess probiotic-related gut microbiome at species-level resolution of the microbiome, coupled with functional evidence and a washout period which adds robustness to any finding of potential signal of treatment effect.

Several limitations in this study should be considered. First, since patients were randomized by class of third antiretroviral drug, same-class ARV drgus might have different effects of the gut microbiome. Additionally, the cohort selected for this study was composed by immunodiscordant individuals, whose low CD4+ counts after viral suppression made them prone to infections that required antibiotic treatment, causing many dropouts. However, no significant biases were created by dropouts among the groups, although final sample size (n=71) was clearly smaller than original target (n=100) and may have been underpowered to detect some effects. Importantly, faecal samples hold an inherent bias and may not be representative of the actual gut microbiome composition, especially from the upper GI. Also, diet and concomitant treatments may affect faecal microbiome composition. While we found low rates of concomitant medications and these were balanced among groups, dietary information was not available, although extreme diets were excluded. This may affect our capability to detect any correlation between clinical outcomes and microbiome features. Finally, the fact that treatment intake was self-reported, could have led to many patients not taking the treatment but reporting otherwise during the 6 months follow-up, thus overestimating the actual intake of the probiotics. All these shortcomings add up to the limited effect of probiotics in immune reconstitution and affect the capability to translate research results from into clinical practice and warrant further research.

## Conclusions

A synbiotic intervention with *L. plantarum* and *P. acidilactici* was safe and well tolerated. Synbiotic intervention led to small increases in CD4/CD8 ratio and minor reductions in sCD14 after 6 months and continued 3 months after discontinuing the intervention, but such changes are of uncertain clinical significance. A probiotic with the same composition but without prebiotics was also safe but did not achieve any impact on immune parameters or faecal microbiome composition.

## Data availability statement

The data presented in the study are deposited in the EBI-ENA repository, accession number PRJEB56022.

## Ethics statement

The study was reviewed and approved by the Institutional Review Board of the Hospital Universitari Germans Trias i Pujol (reference PI-13-046). All participants provided written informed consent in accordance with the World Medical Association Declaration of Helsinki, Fortaleza and Brazil, October 2013 and personal data was managed according to Spanish data protection law (LOPD 15/1999). The study concept, design, patient information and results were discussed with the FLSida Community Advisory Committee, in accordance with AB-Biotics internal QC auditing. All available information can be found in the protocol (**Supplementary Methods**), and the study is registered in clinicaltrials.gov, accession: NCT03542786. The patients/participants provided their written informed consent to participate in this study.

## Author contributions

RP, JE-M, JRS, and MN-J conceived and designed the study. RP and JRS recruited the study participants and performed their clinical evaluations. MA performed study monitoring. MP, AE-T, and MC performed sample processing for sequencing and inflammation markers experiments, under the supervision of MN-J and RP. CB and FC-M performed the bioinformatic and statistical analyses with the supervision of RP, MN-J, CB, RP, and MN-J wrote the paper, which was reviewed, edited, and approved by all authors.
