# Supplementary material for: Probiotic effects on immunity and microbiome in HIV-1 discordant patients
Source: Front Immunol. 2022 Dec 8;13:1066036. doi: 10.3389/fimmu.2022.1066036 (PMC9774487; doi:10.3389/fimmu.2022.1066036)
Supplement: Supplementary file 1 [file DataSheet_1.docx]

**Probiotic/Synbiotic Composition and Manufacturing**


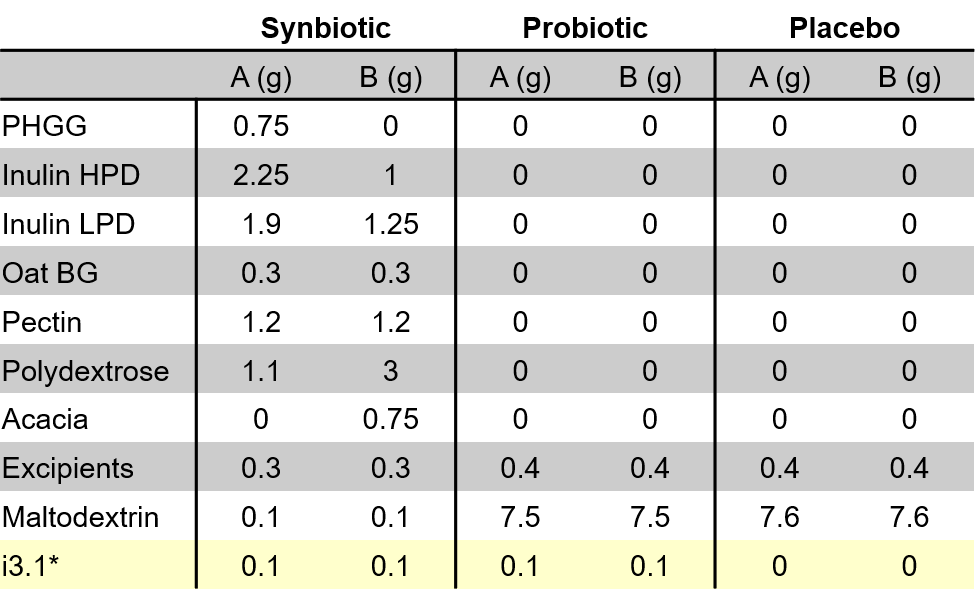
i3.1 is a commercialized and trademarked product. It was provided by the promoter and manufactured in ALIFARM S.A (Polígon Industrial Els Xops, Nau 9, 08185 Lliçà de Vall, Spain), in GMP (Good Manufacturing Practices) certified facility. The product was appropriately labelled as to ensure traceability and the double-blind nature of the study. The following table shows the composition of the i3.1 probiotic final formulation, which is administered as sachets filled with ready-to-dissolve powder. Each participant received the product along with the appropriate instructions to reconstitute the sachets.

* composition of i3.1 corresponds to 10^9^ cfu/sachet of the 3 following strains:

*i3.1 = 3x10^9^ cfu/sachet:

- *Pediococcus acidilactici CECT 7483*
- *Lactobacillus plantarum CECT7484*
- *Lactobacillus plantarum CECT 7485*
- *Pediococcus acidilactici* CECT 7483
- *Lactobacillus plantarum CECT7484*
- *Lactobacillus plantarum* CECT 7485

2 weeks: A

2 weeks: B

Each patient of every arm, was provided the product labelled as either A or B for 15 days, after which it would be switched for the other formulation.
