# Supplementary material for: Probiotic effects on immunity and microbiome in HIV-1 discordant patients
Source: Front Immunol. 2022 Dec 8;13:1066036. doi: 10.3389/fimmu.2022.1066036 (PMC9774487; doi:10.3389/fimmu.2022.1066036)
Supplement: Supplementary file 2 [file DataSheet_2.docx]

**Supplementary table 1:** Distribution of Adverse Events (AEs) across the selected cohort by treatment group, AE category and severity. Only those patients selected for analysis were considered. Counts by class refer to number of patients, while totals count the number of patients who had at least 1 AE per grade and the total number of AE. Patient count and AE counts may differ as some patients may have more than 1 AE of the same grade of severity.

|  |  |  | **Placebo** | **Probiotic** | **Synbiotic** |
| --- | --- | --- | --- | --- | --- |
|  |  |  | *(N=18)* | *(N=21)* | *(N=32)* |
| *Total (Patients)* | | | 9 (50%) | 7 (33.33%) | 17 (53.12%) |
| *Total (Events)* | | | 10 | 11 | 24 |
| **Grade 1** | | |  |  |  |
|  | *Total (Patients)* | | 6 (33.33%) | 5 (23.81%) | 13 (40.62%) |
|  | *Total (Events)* | | 7 | 8 | 14 |
|  |  | Gastrointestinal | 2 | 4 | 6 |
|  |  | Infection | 1 | 0 | 0 |
|  |  | Musculoskeletal | 1 | 0 | 0 |
|  |  | Neurologic | 1 | 0 | 0 |
|  |  | Renal | 0 | 0 | 1 |
|  |  | Respiratory | 0 | 1 | 2 |
|  |  | Skin | 1 | 0 | 4 |
| **Grade 2** | | |  |  |  |
|  | *Total (Patients)* | | 2 (11.11%) | 3 (14.29%) | 7 (21.88%) |
|  | *Total (Events)* | | 2 | 3 | 8 |
|  |  | Gastrointestinal | 0 | 0 | 3 |
|  |  | Infection | 0 | 0 | 1 |
|  |  | Musculoskeletal | 1 | 0 | 1 |
|  |  | Respiratory | 0 | 2 | 1 |
|  |  | Skin | 1 | 1 | 1 |
| **Grade 3** | | |  |  |  |
|  | *Total (Patients)* | | 0 (0%) | 0 (0%) | 1 (3.12%) |
|  | *Total (Events)* | | 0 | 0 | 2 |
|  |  | Gastrointestinal | 0 | 0 | 1 |
| **Grade 4** | | |  |  |  |
|  | *Total (1 event or more)* | | 1 (5.56%) | 0 (0%) | 0 (0%) |
|  | *Total* | | 1 | 0 | 0 |
|  |  | Cancer | 1 | 0 | 0 |

**Supplementary table 2** Comparison of inflammation and permeability markers at baseline. LBP represented as mean-(sd), as it followed a continuous normal distribution (shapiro test pval < 0.05). All other markers are represented as median-[IQR] because of their non-normal continuous distribution. LBP was tested by ANOVA while the remaining ones were tested by Kruskal-Wallis. PI: Protease Inhibitors. Transmission stands for the reported method of transmission of the patients, MSM: Men who have Sex with Men, HTS: heterosexual, IDU: Intravenous Drug Use, other: other forms of transmission, unknown: unknown form of transmission. AIs: Anti-inflammatory intake. PPIs: proton pump inhibitor intake.

|  | **[ALL]** | **Placebo** | **Probiotic** | **Synbiotic** | **p.overall** |  | **N** |
| --- | --- | --- | --- | --- | --- | --- | --- |
|  | ***N=71*** | ***N=18*** | ***N=21*** | ***N=32*** |  |  |  |
| Age | 49.9 (9.36) | 52.8 (10.4) | 49.1 (10.4) | 48.8 (7.88) | 0.316 |  | 71 |
| CD4+ nadir | 124 [74.5;236] | 140 [81.8;212] | 109 [74.0;186] | 157 [70.0;261] | 0.395 |  | 71 |
| Gender: |  |  |  |  | 0.310 |  | 71 |
| F | 10 (14.1%) | 1 (5.56%) | 5 (23.8%) | 4 (12.5%) |  |  |  |
| M | 61 (85.9%) | 17 (94.4%) | 16 (76.2%) | 28 (87.5%) |  |  |  |
| BMI | 24.4 [22.1;25.6] | 24.8 [22.6;26.3] | 24.4 [22.7;25.3] | 24.1 [22.2;25.4] | 0.778 |  | 69 |
| Weight (Kg) | 72.8 (11.0) | 75.2 (12.6) | 72.8 (12.3) | 71.3 (9.08) | 0.491 |  | 69 |
| Height (cm) | 172 (7.13) | 174 (9.26) | 171 (6.98) | 171 (5.71) | 0.336 |  | 71 |
| Transmission |  |  |  |  | 0.485 |  | 71 |
| HTS | 14 (19.7%) | 2 (11.1%) | 6 (28.6%) | 6 (18.8%) |  |  |  |
| IDU | 12 (16.9%) | 4 (22.2%) | 1 (4.76%) | 7 (21.9%) |  |  |  |
| MSM | 38 (53.5%) | 10 (55.6%) | 13 (61.9%) | 15 (46.9%) |  |  |  |
| other | 4 (5.63%) | 1 (5.56%) | 1 (4.76%) | 2 (6.25%) |  |  |  |
| Transfusion | 1 (1.41%) | 1 (5.56%) | 0 (0.00%) | 0 (0.00%) |  |  |  |
| Unknown | 2 (2.82%) | 0 (0.00%) | 0 (0.00%) | 2 (6.25%) |  |  |  |
| Years from diagnosis | 14.0 [6.00;24.0] | 15.5 [7.75;23.8] | 13.0 [6.00;20.0] | 13.5 [4.00;25.0] | 0.603 |  | 71 |
| Years from cART initiation | 0.00 [0.00;2.00] | 0.00 [0.00;4.00] | 0.00 [0.00;2.00] | 0.00 [0.00;2.25] | 0.510 |  | 71 |
| Coinfection (VHC): |  |  |  |  | 0.234 |  | 71 |
| no | 61 (84.5%) | 15 (83.3%) | 20 (95.2%) | 25 (78.1%) |  |  |  |
| yes | 10 (15.5%) | 3 (16.7%) | 1 (4.76%) | 7 (21.9%) |  |  |  |
| Third drug class |  |  |  |  | 0.282 |  | 71 |
| INSTI | 47 (66.2%) | 9 (50.0%) | 16 (76.2%) | 22 (68.8%) |  |  |  |
| NNRTI | 18 (25.4%) | 6 (33.3%) | 5 (23.8%) | 7 (21.9%) |  |  |  |
| PI | 6 (8.45%) | 3 (16.7%) | 0 (0.00%) | 3 (9.38%) |  |  |  |
| AIs |  |  |  |  | 1.000 |  | 71 |
| No | 67 (94.4%) | 17 (94.4%) | 20 (95.2%) | 30 (93.8%) |  |  |  |
| yes | 4 (5.63%) | 1 (5.56%) | 1 (4.76%) | 2 (6.25%) |  |  |  |
| PPIs |  |  |  |  | 0.254 |  | 71 |
| No | 70 (98.6%) | 17 (94.4%) | 21 (100%) | 32 (100%) |  |  |  |
| Yes | 1 (1.41%) | 1 (5.56%) | 0 (0.00%) | 0 (0.00%) |  |  |  |
| LBP (μg/mL) | 5.99 (2.17) | 5.71 (2.31) | 6.12 (2.54) | 6.06 (1.86) | 0.815 | 70 |  |
| sCD14 (μg/mL) | 2.48 [2.01;2.92] | 2.62 [2.03;2.95] | 2.62 [2.03;3.25] | 2.36 [1.90;2.61] | 0.087 | 70 |  |
| IL6 (pg/ml) | 0.95 [0.65;1.72] | 0.87 [0.59;2.41] | 0.74 [0.64;1.67] | 1.02 [0.68;1.42] | 0.934 | 70 |  |
| CRP (μg/mL) | 18.1 [10.0;64.5] | 17.5 [9.14;48.0] | 15.3 [13.4;56.9] | 20.3 [10.4;65.6] | 0.901 | 70 |  |
| D-Dimer (μg/mL) | 2.76 [2.14;3.69] | 2.67 [2.02;3.96] | 2.56 [1.97;3.48] | 2.76 [2.39;4.02] | 0.290 | 70 |  |
| CD4+ (counts/ml) | 397 [337;466] | 358 [344;427] | 403 [318;466] | 422 [338;476] | 0.259 | 68 |  |
| CD88+ (counts/ml) | 696 [507;870] | 734 [603;819] | 690 [507;877] | 657 [500;865] | 0.583 | 68 |  |
| CD4/CD8 | 0.55 [0.42;0.83] | 0.49 [0.42;0.61] | 0.56 [0.40;0.67] | 0.57 [0.45;0.88] | 0.333 | 68 |  |

**Supplementary table 3:** Numbers of patients selected at any timepoint for across-group comparisons for every variable. Separated by arm and probiotic detection. Number of patients may differ at different variables for the same group/timepoint due to lack of sample/missing data which may be otherwise available for other variables.

|  | month | Placebo | Probiotic | Synbiotic | Absent | Present |
| --- | --- | --- | --- | --- | --- | --- |
| LBP | 0 | 18 | 21 | 31 | 36 | 16 |
|  | 3 | 15 | 17 | 31 | 32 | 16 |
|  | 6 | 15 | 19 | 27 | 32 | 14 |
|  | 9 | 12 | 17 | 21 | 25 | 13 |
| sCD14 | 0 | 18 | 21 | 31 | 36 | 16 |
|  | 3 | 15 | 17 | 31 | 32 | 16 |
|  | 6 | 15 | 19 | 27 | 32 | 14 |
|  | 9 | 12 | 17 | 21 | 25 | 13 |
| IL6 | 0 | 18 | 21 | 31 | 36 | 16 |
|  | 3 | 15 | 17 | 31 | 32 | 16 |
|  | 6 | 15 | 19 | 27 | 32 | 14 |
|  | 9 | 12 | 17 | 21 | 25 | 13 |
| CRP | 0 | 18 | 21 | 31 | 36 | 16 |
|  | 3 | 15 | 17 | 31 | 32 | 16 |
|  | 6 | 15 | 19 | 27 | 32 | 14 |
|  | 9 | 12 | 17 | 21 | 25 | 13 |
| DDimer | 0 | 18 | 21 | 31 | 36 | 16 |
|  | 3 | 15 | 17 | 31 | 32 | 16 |
|  | 6 | 15 | 19 | 27 | 32 | 14 |
|  | 9 | 12 | 17 | 21 | 25 | 13 |
| CD4 | 0 | 18 | 21 | 31 | 36 | 16 |
|  | 3 | 15 | 17 | 31 | 32 | 16 |
|  | 6 | 15 | 19 | 27 | 32 | 14 |
|  | 9 | 12 | 17 | 21 | 25 | 13 |
| CD8 | 0 | 18 | 21 | 31 | 36 | 16 |
|  | 3 | 15 | 17 | 31 | 32 | 16 |
|  | 6 | 15 | 19 | 27 | 32 | 14 |
|  | 9 | 12 | 17 | 21 | 25 | 13 |
| CD4_8  ratio | 0 | 18 | 21 | 31 | 36 | 16 |
|  | 3 | 15 | 17 | 31 | 32 | 16 |
|  | 6 | 15 | 19 | 27 | 32 | 14 |
|  | 9 | 12 | 17 | 21 | 25 | 13 |

**Suplementary table 4:** Numbers of patients at any timepoint for longitudinal withing-group comparisons in every variable, column “months” states which timepoints have been compared. The number of patients for the same group/variable may differ due to the comparisons being paired, which requires all patients to data at both timepoints, which may cause some patients to be discarded, changing the numbers from supplementary table 2.

|  | **months** | **Placebo** | **Probiotic** | **Synbiotic** | **Absent** | **Present** |
| --- | --- | --- | --- | --- | --- | --- |
| **sCD14** | 0-3 | 15 | 17 | 30 | 31 | 16 |
|  | 0-6 | 15 | 19 | 26 | 31 | 14 |
|  | 0-9 | 12 | 17 | 20 | 24 | 13 |
|  | 3-6 | 14 | 16 | 26 | 28 | 14 |
|  | 3-9 | 11 | 15 | 21 | 23 | 13 |
|  | 6-9 | 12 | 17 | 20 | 24 | 13 |
| **LBP** | 0-3 | 15 | 17 | 30 | 31 | 16 |
|  | 0-6 | 15 | 19 | 26 | 31 | 14 |
|  | 0-9 | 12 | 17 | 20 | 24 | 13 |
|  | 3-6 | 14 | 16 | 26 | 28 | 14 |
|  | 3-9 | 11 | 15 | 21 | 23 | 13 |
|  | 6-9 | 12 | 17 | 20 | 24 | 13 |
| **IL6** | 0-3 | 15 | 17 | 30 | 31 | 16 |
|  | 0-6 | 15 | 19 | 26 | 31 | 14 |
|  | 0-9 | 12 | 17 | 20 | 24 | 13 |
|  | 3-6 | 14 | 16 | 26 | 28 | 14 |
|  | 3-9 | 11 | 15 | 21 | 23 | 13 |
|  | 6-9 | 12 | 17 | 20 | 24 | 13 |
| **CRP** | 0-3 | 15 | 17 | 30 | 31 | 16 |
|  | 0-6 | 15 | 19 | 26 | 31 | 14 |
|  | 0-9 | 12 | 17 | 20 | 24 | 13 |
|  | 3-6 | 14 | 16 | 26 | 28 | 14 |
|  | 3-9 | 11 | 15 | 21 | 23 | 13 |
|  | 6-9 | 12 | 17 | 20 | 24 | 13 |
| **DDimer** | 0-3 | 15 | 17 | 30 | 31 | 16 |
|  | 0-6 | 15 | 19 | 26 | 31 | 14 |
|  | 0-9 | 12 | 17 | 20 | 24 | 13 |
|  | 3-6 | 14 | 16 | 26 | 28 | 14 |
|  | 3-9 | 11 | 15 | 21 | 23 | 13 |
|  | 6-9 | 12 | 17 | 20 | 24 | 13 |
| **CD4** | 0-3 | 14 | 16 | 28 | 28 | 16 |
|  | 0-6 | 13 | 17 | 21 | 24 | 14 |
|  | 0-9 | 12 | 16 | 19 | 23 | 12 |
|  | 3-6 | 13 | 13 | 23 | 22 | 14 |
|  | 3-9 | 11 | 13 | 21 | 22 | 12 |
|  | 6-9 | 11 | 15 | 20 | 23 | 12 |
| **CD8** | 0-3 | 14 | 16 | 28 | 28 | 16 |
|  | 0-6 | 13 | 17 | 21 | 24 | 14 |
|  | 0-9 | 12 | 16 | 19 | 23 | 12 |
|  | 3-6 | 13 | 13 | 23 | 22 | 14 |
|  | 3-9 | 11 | 13 | 21 | 22 | 12 |
|  | 6-9 | 11 | 15 | 20 | 23 | 12 |
| **CD4_8_ratio** | 0-3 | 14 | 16 | 28 | 28 | 16 |
|  | 0-6 | 13 | 17 | 21 | 24 | 14 |
|  | 0-9 | 12 | 16 | 19 | 23 | 12 |
|  | 3-6 | 13 | 13 | 23 | 22 | 14 |
|  | 3-9 | 11 | 13 | 21 | 22 | 12 |
|  | 6-9 | 11 | 15 | 20 | 23 | 12 |


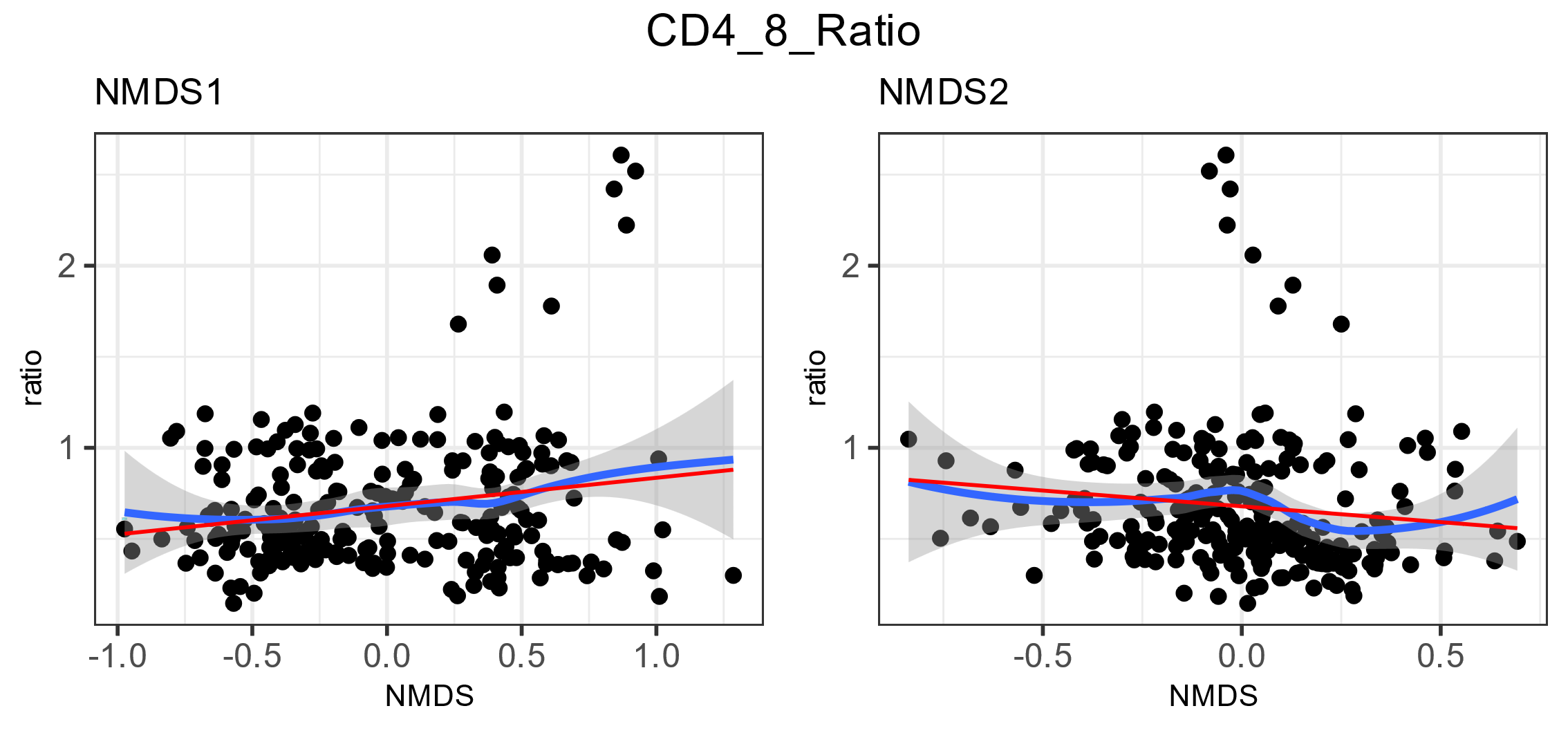


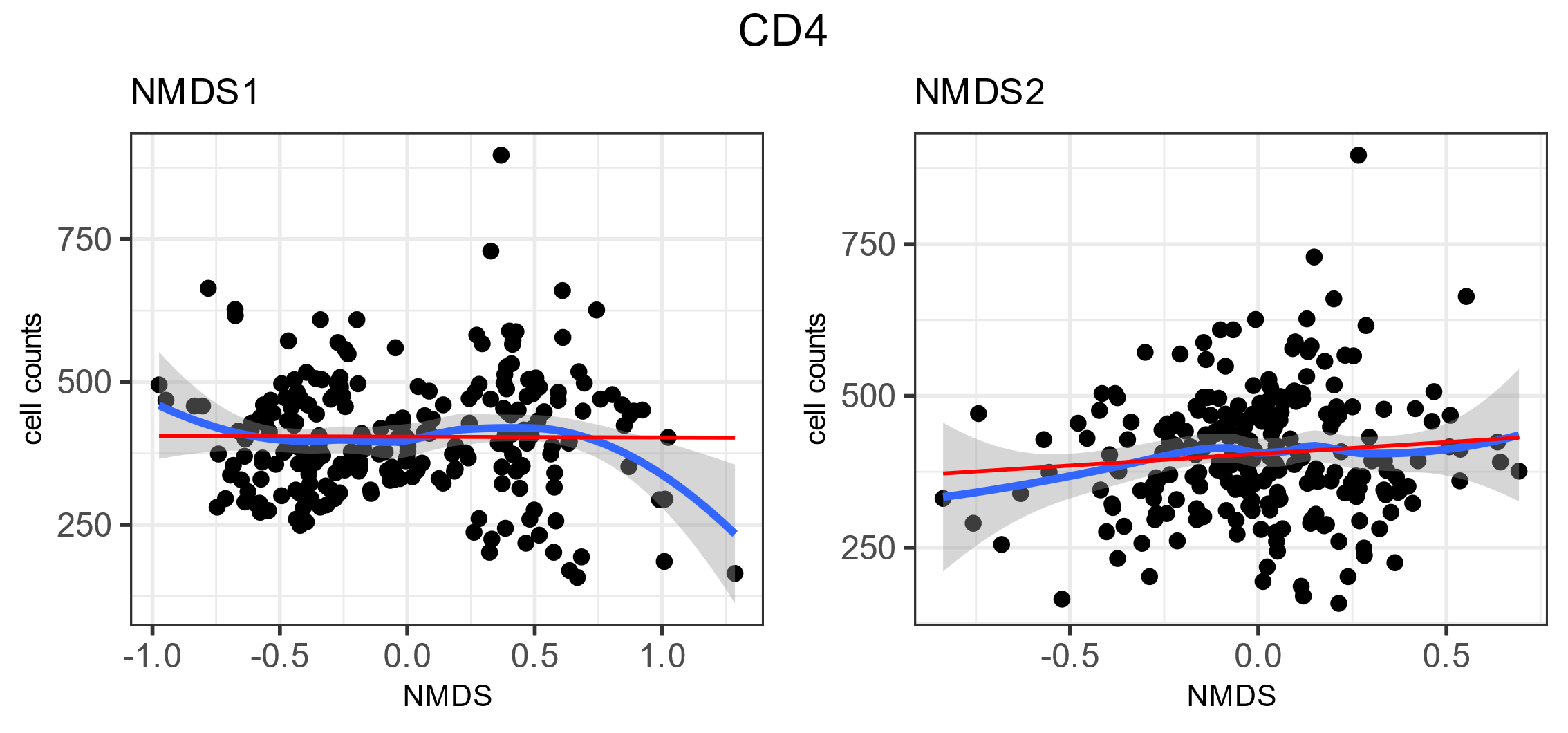

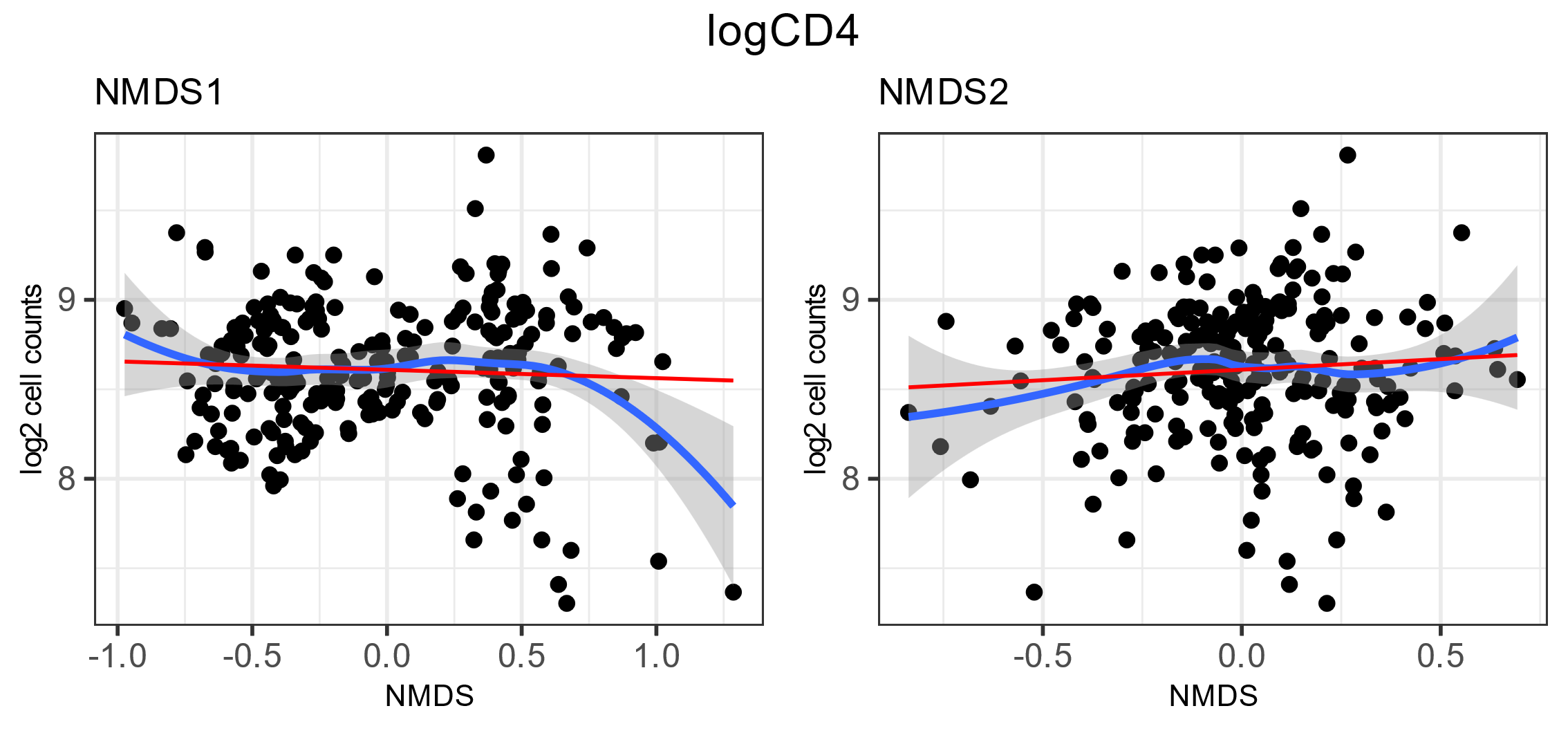

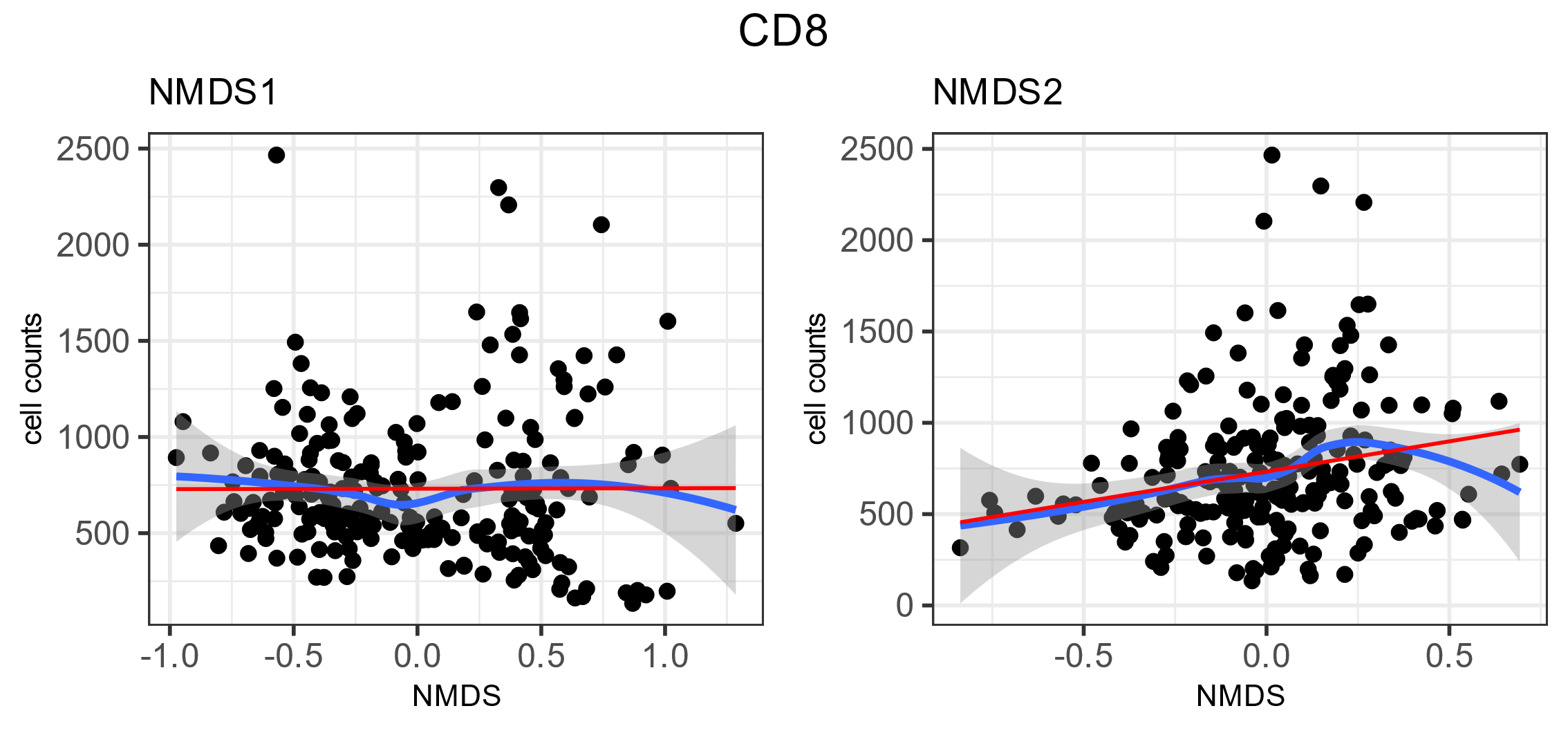

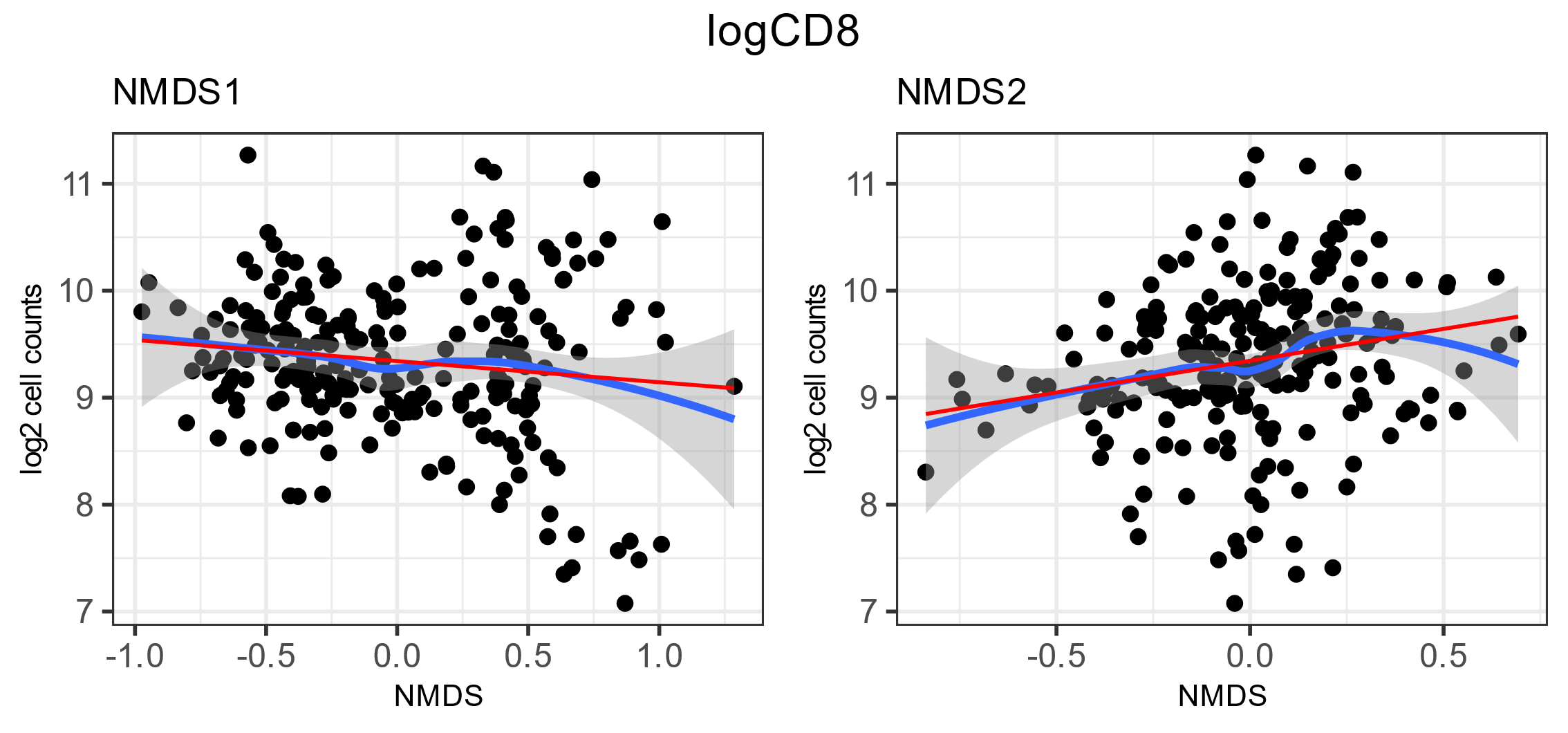

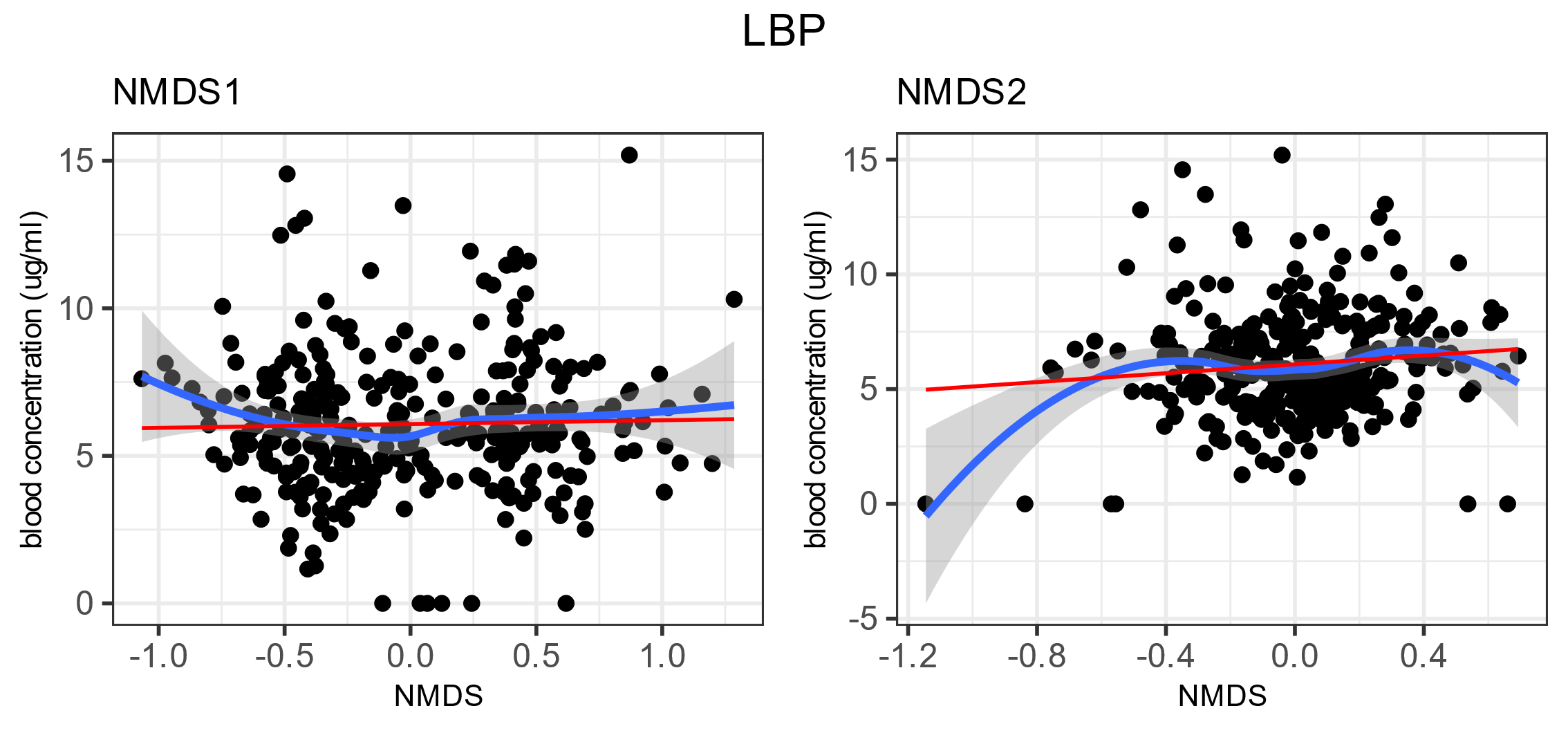


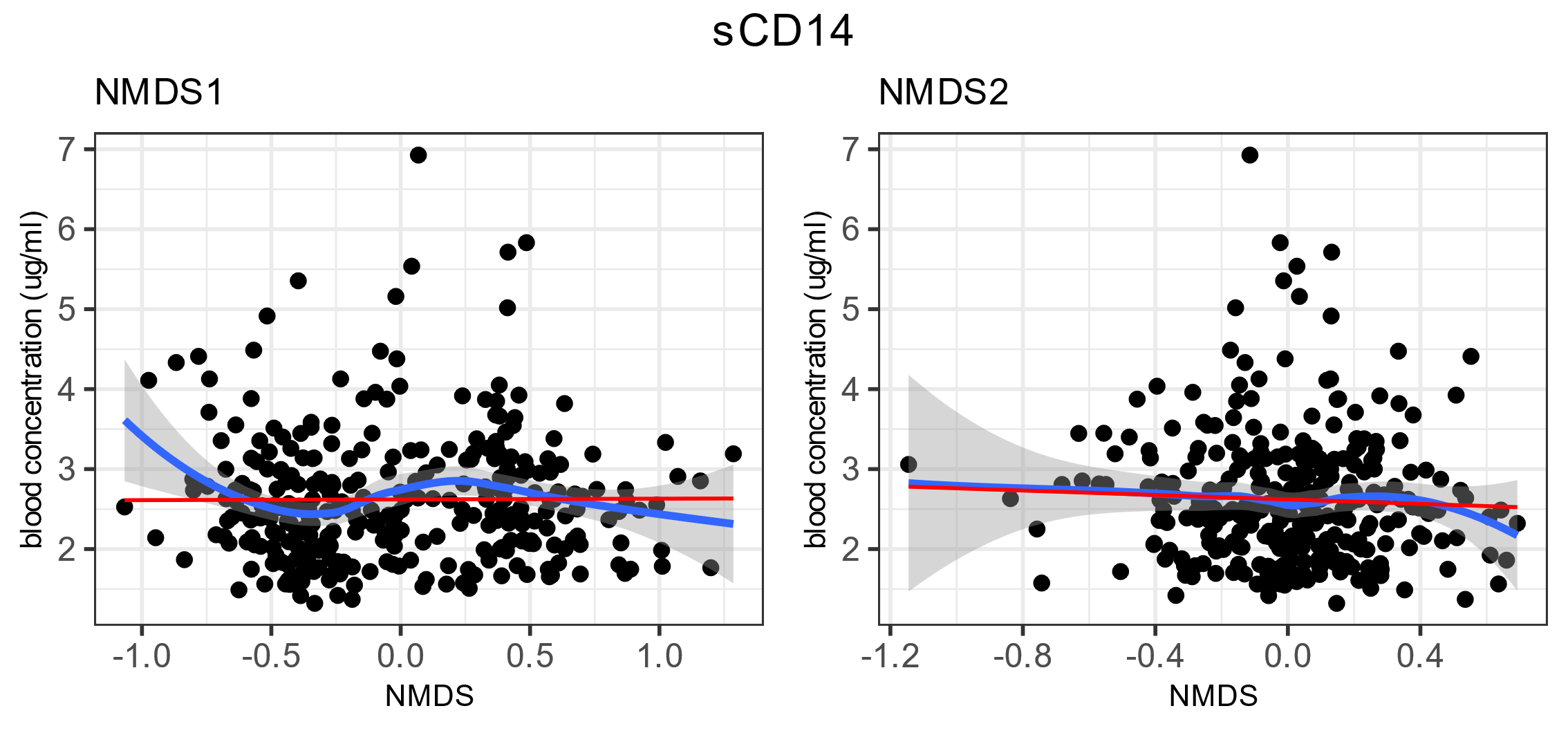


**Supplementary figure 1**: Correlation between microbiota and the selected clinical variables. Data is represented as scatterplots and complemented by a loess regression with confidence intervals (blue line, grey smooth) and a simple linear model (red). The fact that most variables head a somewhat good agreement between both fits, along with all of them showing monotonicity was taken as proof that linear correlation could be performed.

**Supplementary table 5**: Correlations for every variable against each NMDS as obtained by envfit function (Top) and from basic spearman correlation (bottom).

|  | NMDS1 | | NMDS2 | |
| --- | --- | --- | --- | --- |
|  | rho | p | rho | p |
| CD4 | 0.045 | 0.496 | 0.045 | 0.498 |
| CD8 | -0.101 | 0.125 | **0.250** | **0.000** |
| LBP | 0.009 | 0.874 | 0.107 | 0.059 |
| sCD14 | 0.018 | 0.754 | -0.043 | 0.453 |
| logCD4 | 0.045 | 0.496 | 0.045 | 0.498 |
| logCD8 | -0.101 | 0.125 | **0.250** | **0.000** |
| CDRatio | 0.071 | 0.279 | **-0.234** | **0.000** |


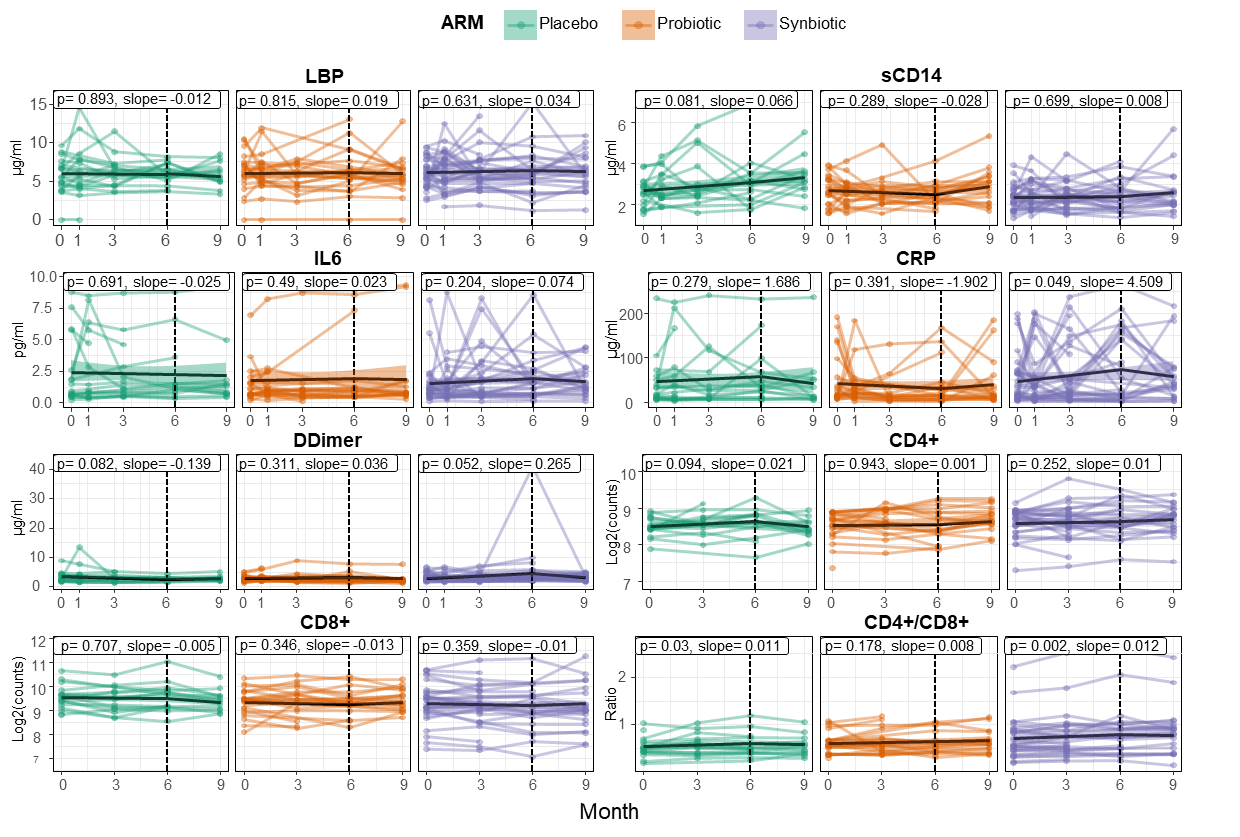


**Supplementary figure 2:** Linear Mixed Models (LMMs) of different clinical variables over time, separated by treatment group. Time and group were selected as fixed effects with patient ID as random. Models are split around month 6 as it marks the start of the washout period.


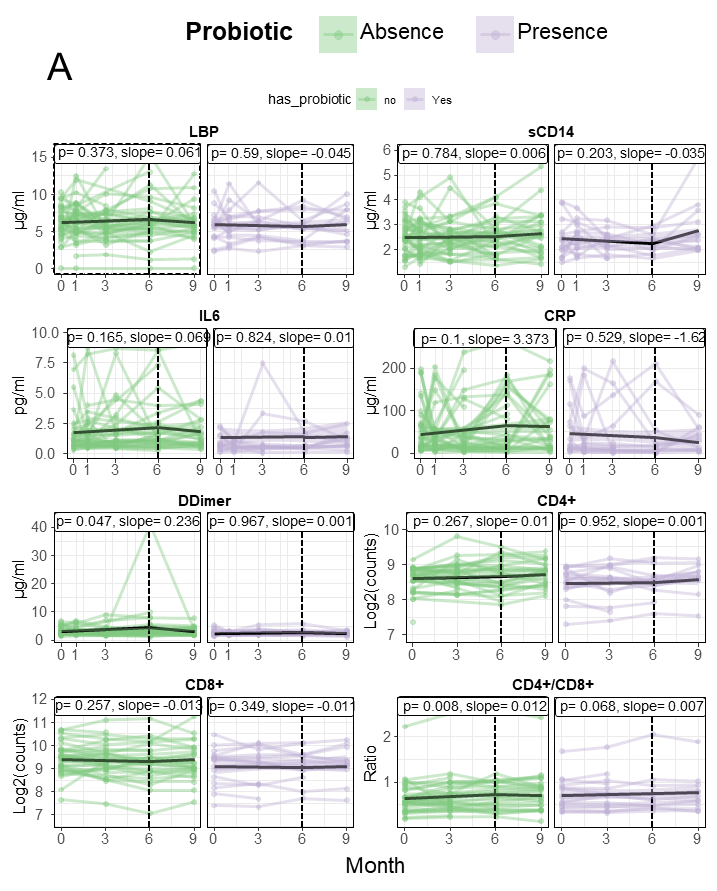


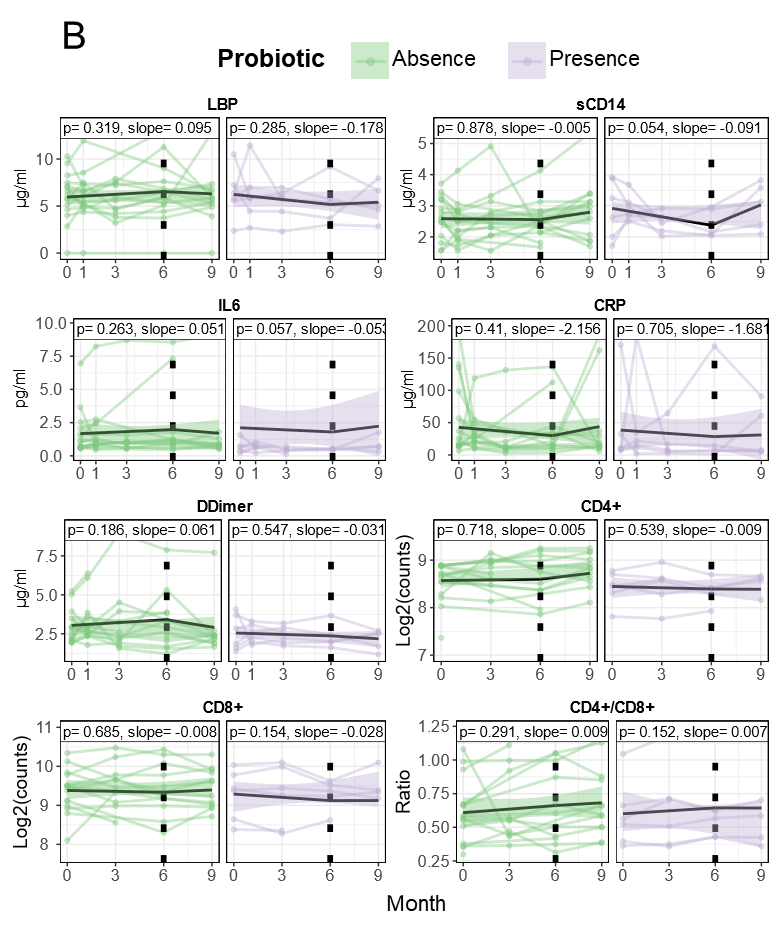


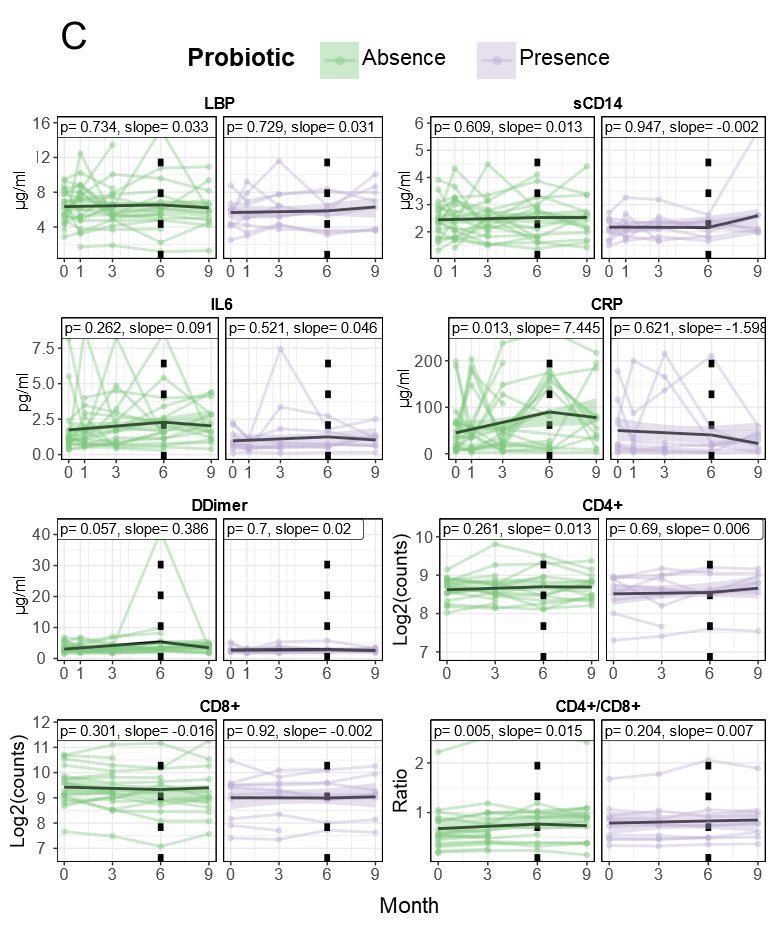


**Supplementary figure 3:**  Evolution over time of different clinical variables (by LMM) by probiotic presence in those patients who received treatment (Placebo excluded,) and split between Probiotic (B) and Synbiotic (C).


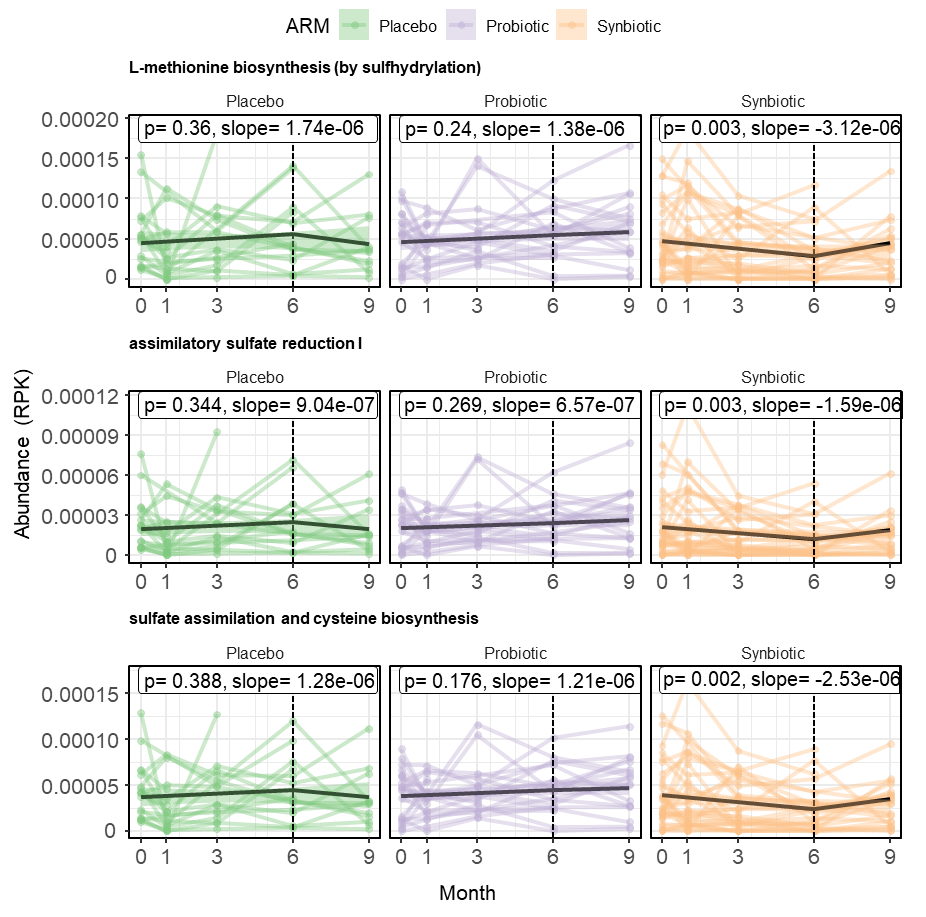


**Supplementary figure 4:** Linear Mixed Models (LMMs) modelling the longitudinal evolution of detected gene pathways over time, separated by intervention. Time and group were selected as fixed effects with patient ID as random. Models are split around month 6 as it marks the start of the washout period.


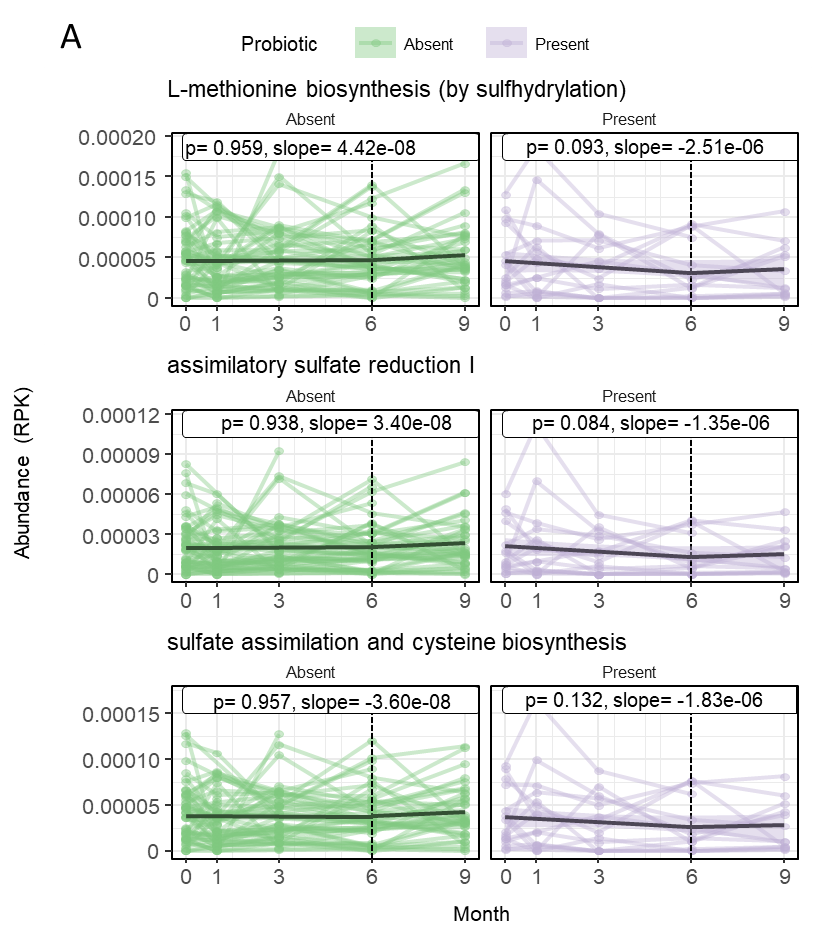


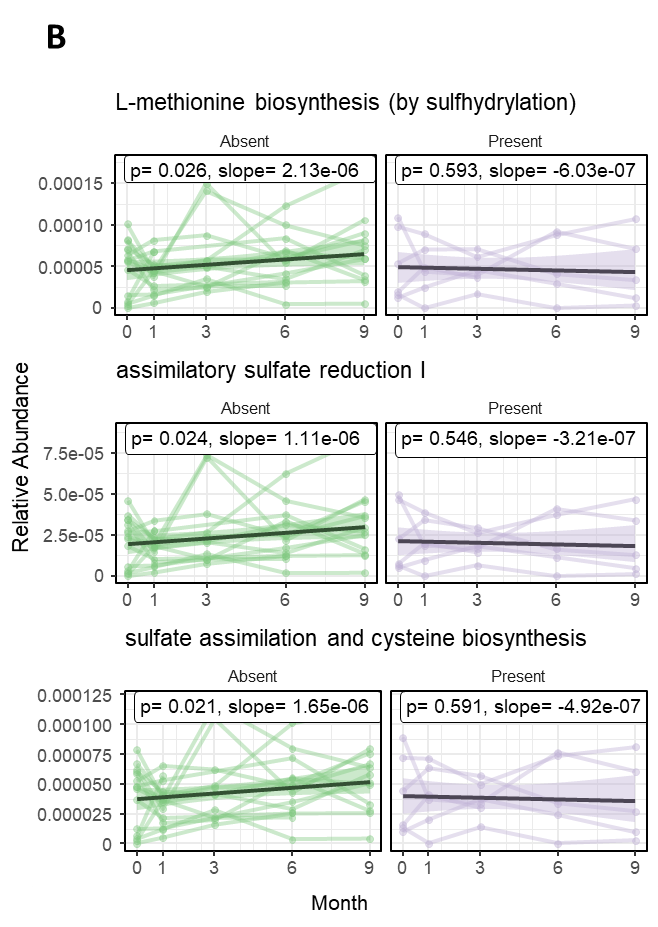


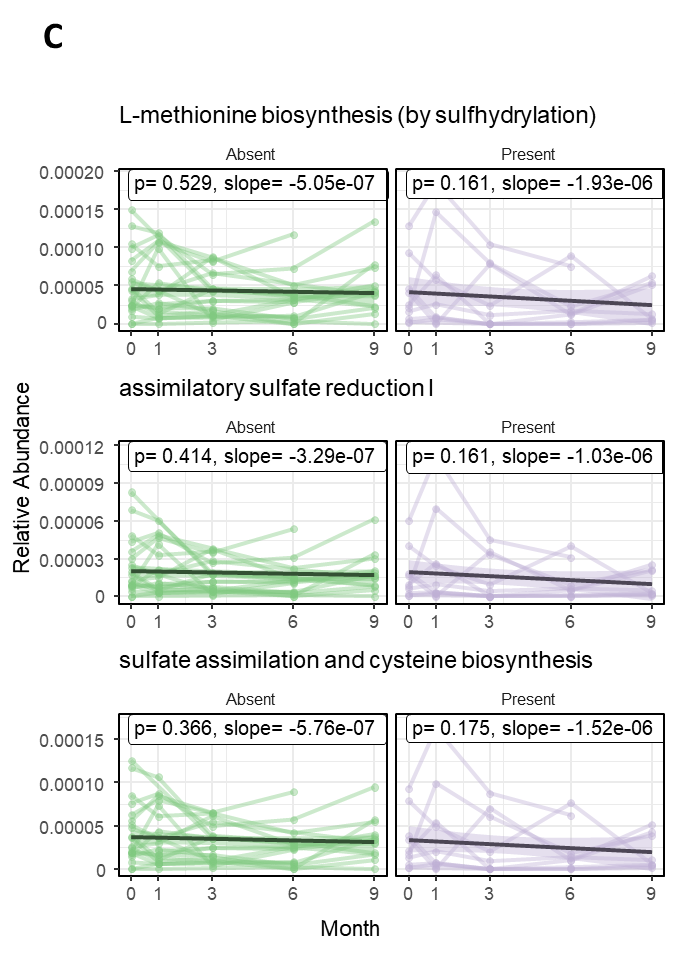


**Supplementary figure 5:**  Evolution over time of different clinical variables (by LMM) by probiotic presence in those patients who received treatment (Placebo excluded, C) and split between Probiotic (B) and Synbiotic (C).


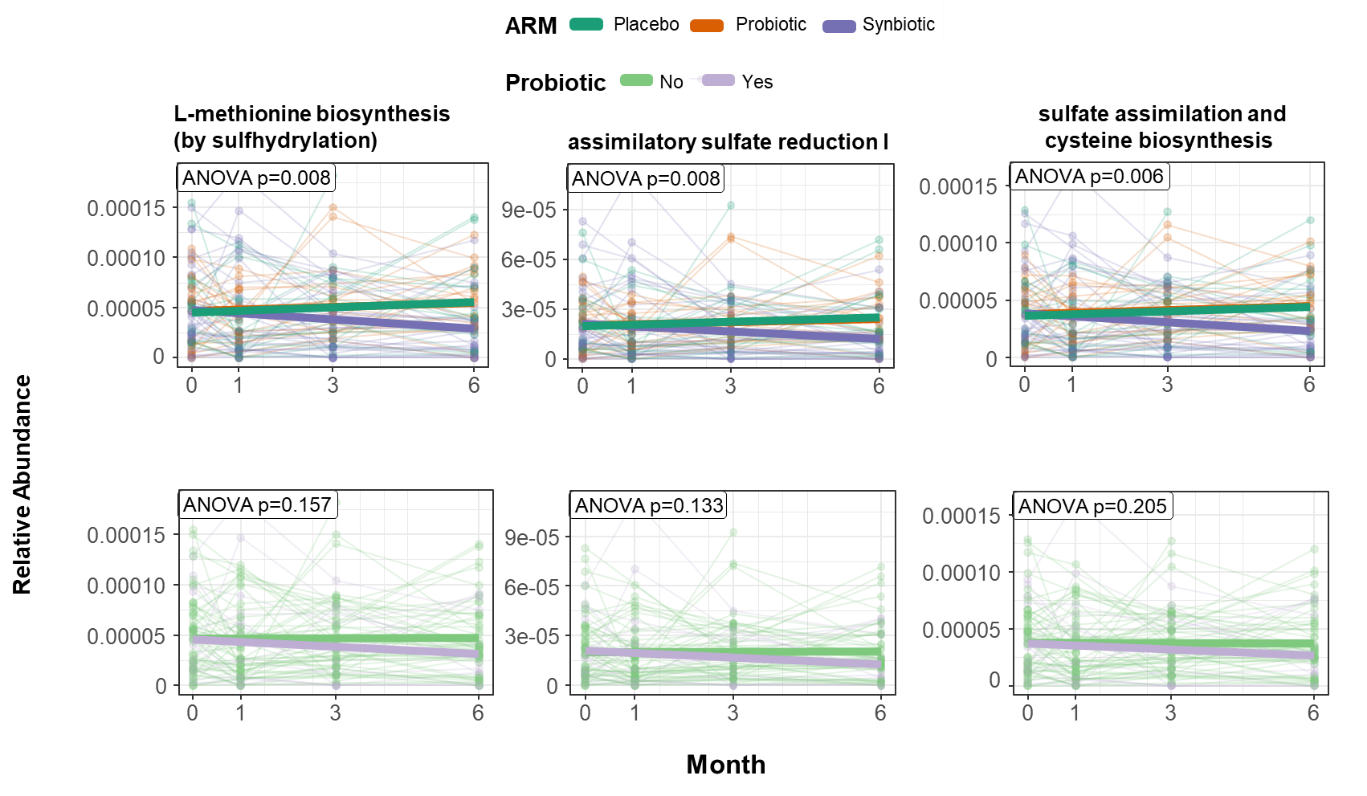


A

B


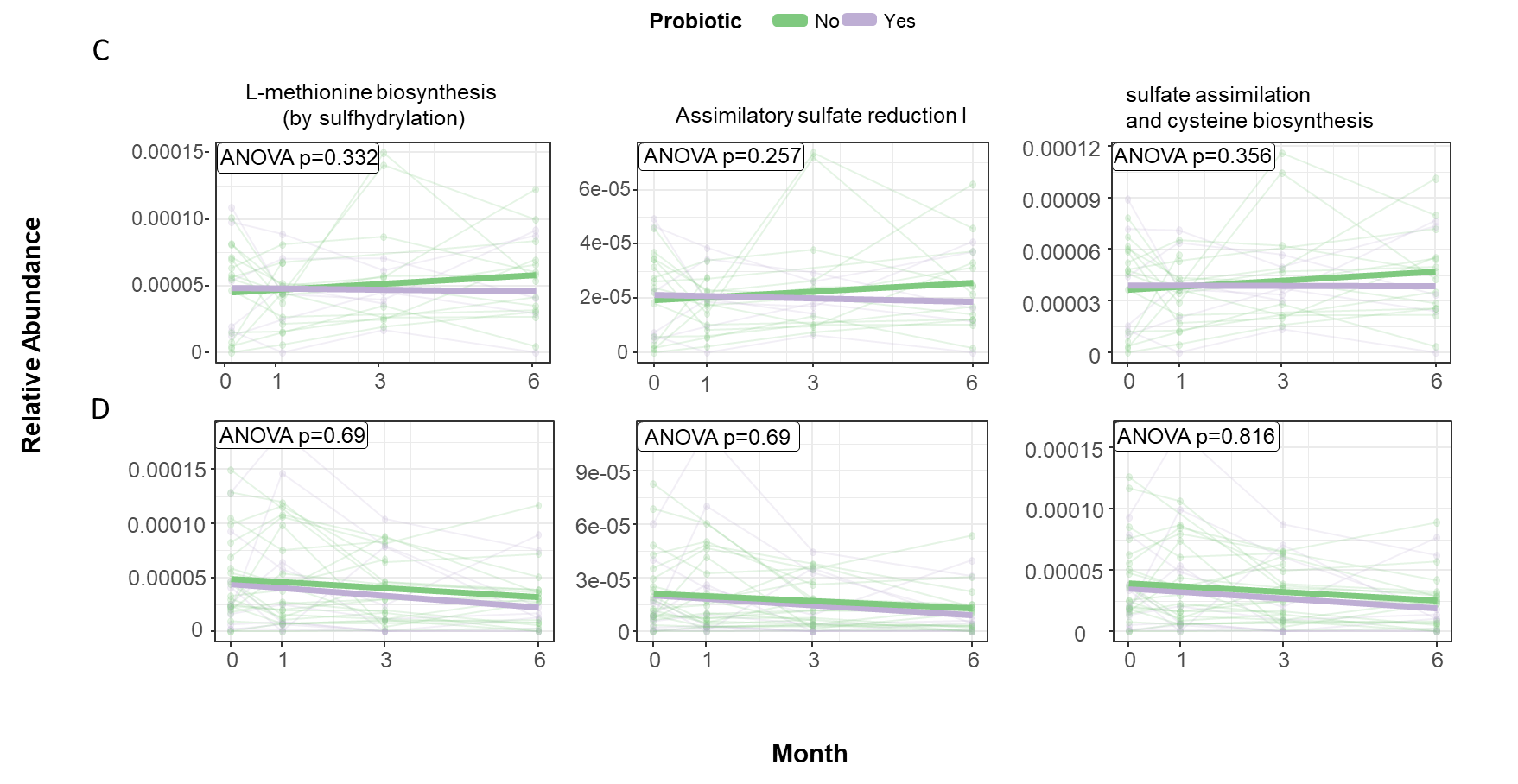


**Supplementary figure 6:** Longitudinal evolution off detected differentially abundant gene pathways per treatment group (A), probiotic presence (B) and probiotic presence split between those in the probiotic (C) and synbiotic (D) groups.
